# Supplementary material for: Human macula formation involves two waves of retinoic acid suppression via CYP26A1 that modulate cell cycle exit and cone subtype specification
Source: Stem Cell Reports. 2025 Sep 25;20(10):102647. doi: 10.1016/j.stemcr.2025.102647 (PMC12790723; doi:10.1016/j.stemcr.2025.102647)
Supplement: Document S2. Article plus supplemental information [file mmc2.pdf]

Stem Cell Reports
Article
OPEN ACCESS

Human macula formation involves two waves of retinoic acid suppression via *CYP26A1* that modulate cell cycle exit and cone subtype specification

Philippa Harding,[1,*] Maja Wojtynska,[1] Alexander J. Smith,[1] Robin R. Ali,[1] and Rachael A. Pearson[1,2,*]
[1]Ocular Cell and Gene Therapy Group, Centre for Gene Therapy and Regenerative Medicine, King's College London, Tower Wing, Guy's Hospital, London SE1 9RT, UK
[2]Lead contact
*Correspondence: philippa.harding@kcl.ac.uk (P.H.), rachael.pearson@kcl.ac.uk (R.A.P.)
https://doi.org/10.1016/j.stemcr.2025.102647

SUMMARY

The human macula is a specialized, cone-rich region of the eye, critical for high-acuity vision, yet the pathways regulating its development remain poorly understood. RA-catabolizing enzyme *CYP26A1* establishes the chick high-acuity area via upregulation of fibroblast growth factor 8 (*FGF8*). However, detailed analysis of this pathway and its functions has not been performed in early human fetal tissue. Fluorescent *in situ* hybridization revealed striking biphasic *CYP26A1* expression but little *FGF8* in the presumptive macula region between post-conception weeks (PCW) 6–17. Pharmacological retinoic acid (RA) signaling inhibition in human retinal organoids mimicking the two waves of *CYP26A1* revealed early RA inhibition prompted early cell cycle exit and increased cone genesis, while late inhibition altered cone subtype specification. Conversely, recombinant FGF8 had no effect on photoreceptor fate. This work provides spatiotemporal examination of *CYP26A1* across human macular development, as well as experimental evidence for the different roles of RA signaling inhibition in a human model of retinal development.

INTRODUCTION

The macula is a 5–6 mm region at the center of the retina, containing within it the fovea, an area critical for high-acuity and color vision in humans and simian primates (Hoshino et al., 2017; Voigt et al., 2021). Damage and degeneration in this region lead to debilitating sight loss, significantly impacting activities such as reading and recognizing faces. The macula has a unique architecture comprising a high density of cone photoreceptors, contrasting with the rod-dominant peripheral retina, the highest density (200,000 cones/mm^2) being at the center of the macula in the foveola, a 300 μm avascular rod-free zone (RFZ) consisting exclusively of medium (M/green) and long (L/red) wavelength-detecting cones (Curcio et al., 1990; Voigt et al., 2021). The proportion of cones: rods declines with increasing eccentricity, being ~1:1 in the fovea, dropping to 1:30 in the peripheral retina. Additionally, the macula contains the highest density of retinal ganglion cells (RGCs) in the retina, and a highly ordered synaptic configuration to aid rapid phototransduction.

The retina exhibits a striking center-to-periphery gradient of development, the central retina entering neurogenesis ~50 days before peripheral regions (Hoshino et al., 2017), while macular differentiation precedes even the equivalent region in the nasal retina, with immature cones present in the presumptive macula (PM) by the end of post-conception week (PCW)8/Carnegie stage (CS)23 (Hendrickson and Zhang, 2019). However, due to central migration of cones into the fovea, adult cone density is not achieved until 4–6 years (Curcio et al., 1990). Despite their importance, the molecular mechanisms underlying human macular and foveal development remain poorly understood, owing to the lack of equivalent structures in classical animal models. Furthermore, minimal evaluation of macular formation has been performed in human tissue prior to PCW8, due to limited tissue samples, and the lack of a macular marker expressed at this early stage (Hendrickson, 2016). Some other species do possess a broadly analogous high-acuity region; chickens possess an RFZ, called the *area centralis* or high-acuity area (HAA) (Mey and Thanos, 2000). Recent research has shown that chick HAA formation is dependent on regulation of retinoic acid (RA) levels (Silva and Cepko, 2017). RA is a morphogen that can control gene expression through binding to RA receptors (RARs) and retinoid X receptors, which then engage RA-response elements (RAREs) (Cvekl and Wang, 2009). Upon binding to RAREs, co-activators are recruited and lead to transcriptional regulation of specific RA-regulated genes (Cunningham and Duester, 2015). RA is synthesized by retinal dehydrogenases (ALDHA1–3) and catabolized by cytochrome P450 family 26 enzymes (CYP26s) (Cvekl and Wang, 2009).

In the developing chick retina, strong expression of *Cyp26a1/c1* was observed at a single spot at the presumptive HAA early, coincident and colocalized with similarly strong expression of fibroblast growth factor 8 (*Fgf8*), which is otherwise downregulated by RA (Cunningham and Duester, 2015; Silva and Cepko, 2017). Accordingly, transient addition of RA led to loss of *Fgf8* expression, and disruption of the RFZ and a decrease in the high RGC density typical of the HAA (Silva and Cepko, 2017). In

Published by Elsevier Inc. on behalf of International Society for Stem Cell Research.
This is an open access article under the CC BY license (http://creativecommons.org/licenses/by/4.0/).

the same study, expression of *CYP26A1* mRNA was observed in the human PM at CS22 (late PCW8), indicating a potentially conserved signaling pathway in HAA/macula development. In support of this, increased expression of *Cyp26a1* by Müller glia (MG) has been observed, albeit much later in development, in the zebrafish HAA (Lahne et al., 2023), macaque, and marmoset (Peng et al., 2019; Krueger et al., 2024), and bulk and single-cell RNA sequencing (RNA-seq) studies have reported *CYP26A1* to be significantly upregulated in human macula compared with peripheral retina in mid-gestation (PCW20) and in the MG of adult retina human retina (Peng et al., 2019; Cowan et al., 2020). Notably, recent detailed analysis of *CYP26A1* and *FGF8* expression in macaque (Krueger et al., 2024) found that *CYP26A1*, but not *FGF8*, is highly expressed in the developing macular region at day 40, after the onset of cone genesis (corresponding to human ~ PCW10 [Hendrickson and Zhang, 2019]). However, little is known about the spatiotemporal expression of *CYP26A1* and *FGF8* in human fetal retina across the whole period of macular development. Moreover, while various roles have been ascribed to RA signaling across retinal development, the precise downstream functions of RA inhibition in human macular formation are not known.

Human stem cell-derived retinal organoids (hROs) broadly resemble the human neural retina and closely follow the timing of human retinal development (Gonzalez-Cordero et al., 2017). As such, they provide an opportunity to explore the signaling pathways that regulate human retinal development. Here, we used *in situ* hybridization in human fetal tissue and pharmacological manipulation in hROs to investigate the spatiotemporal expression and potential roles of RA and *FGF8* in human macular development.

RESULTS

The RA catabolizing enzyme *CYP26A1* is expressed in two distinct waves within the developing human macula

To determine the spatiotemporal expression of *CYP26A1* and *FGF8*, we performed quantitative RNAScope *in situ* hybridization on human fetal retina samples between PCW6 (CS16-17) and PCW17 (Figure 1). No distinct labeling for *CYP26A1* was visible anywhere in the retina at PCW6, but at PCW7 (CS18-19) weak, specific expression was just visible and localized to a single spot approximately 1 mm temporal to the optic nerve head (ONH) (Figure 1A), a location identified previously as the PM (Hendrickson, 2016). Within this region, *CYP26A1* was distributed throughout the neuroblastic layer (NBL), colocalizing with VSX2$^+$ retinal progenitor cells (RPCs) (Figure 1Bi).

At the start of PCW8 (CS20-21, herein termed "early PCW8"), significantly higher levels of *CYP26A1* were observed in the PM within the VSX2$^+$ NBL, compared to PCW7 ($q(15) = 4.79$, $p = 0.05$; Figures 1A, 1Bi, and 1Ci), with expression significantly reducing again by the end of PCW8 (CS22-23, herein termed "late PCW8"; $q(15) = 6.34$, $p = 0.006$; Figures 1A, 1Bi, and 1Ci). *CYP26A1* was also expressed in the lens epithelium and at low levels in the nasal edge of the retina and around the ONH at PCW7-early PCW8 (Figures 1A and S1A). To provide context regarding retinal maturation, at late PCW8 RPCs expressing the cone/bipolar progenitor marker OTX2 were distributed sparsely throughout the NBL of the entire retina, but were more densely concentrated in the PM, where they were located toward the apical side of the retina (Figures 2Ai–2Aiv). The RXRγ$^+$ nascent ganglion cell layer (GCL) was visible throughout the retina, but only a few RECOVERIN$^+$/RXRγ$^+$ cone photoreceptor cells were visible, in the PM (Figures 2Ai and 2Aii).

By PCW10, *CYP26A1* mRNA expression was significantly reduced in the PM region, compared to early PCW8 ($q(15) = 7.28$, $p = 0.002$; Figures 1A, 1Bi, and 1Ci); moreover, its expression was restricted to a band of VSX2$^+$/OTX2$^+$ cells located in the nascent inner nuclear layer (INL) (Figures 2Bi–2Biv), At PCW10, RXRγ$^+$/RECOVERIN$^+$/OTX2$^+$ cone photoreceptors were located exclusively within a single cell layer (the nascent outer nuclear layer [ONL]) at the apical edge of the PM, which did not express *CYP26A1* (Figures 2Bi–2Biv). RXRγ$^+$/RECOVERIN$^+$ cones were not present in the peripheral retina at this stage (Figure 2Bi).

At PCW12, *CYP26A1* expression was visibly increased within the PM, compared with PCW10, localizing to the basal edge of the VSX2$^+$ INL, although this was not statistically significant (Figures 1A, 1Bi, and 1Ci). However, by PCW17, expression of *CYP26A1* was significantly higher than PCW10 ($q(15) = 9.71$, $p < 0.0001$; Figures 1A, 1Bi, and 1Ci) and PCW12 ($q(15) = 7.23$, $p = 0.002$; Figures 1A, 1Bi, 1Ci, and S1C). At this stage, *CYP26A1* expression was exclusively within the PM (Figure 1A) and almost entirely restricted to VSX2$^+$/SOX9$^+$ MG cells, with minimal signal visible in the VSX2$^+$/SOX9$^-$ bipolar cells or the ONL (Figure 2C).

Spatiotemporal *FGF8* expression pattern is not correlated with the human PM

In the chick, *Fgf8* is initially expressed in a comet-like pattern, with a broad stripe of lower expression extending temporally and a strong, central spot of expression overlapping with the spot-like expression of *Cyp26a1/c1* within the HAA (Silva and Cepko, 2017). In human fetal retina, positive *FGF8* mRNA labeling was visible around the ONH throughout PCW6–17 (Figures 1A, S1A, and S1B),

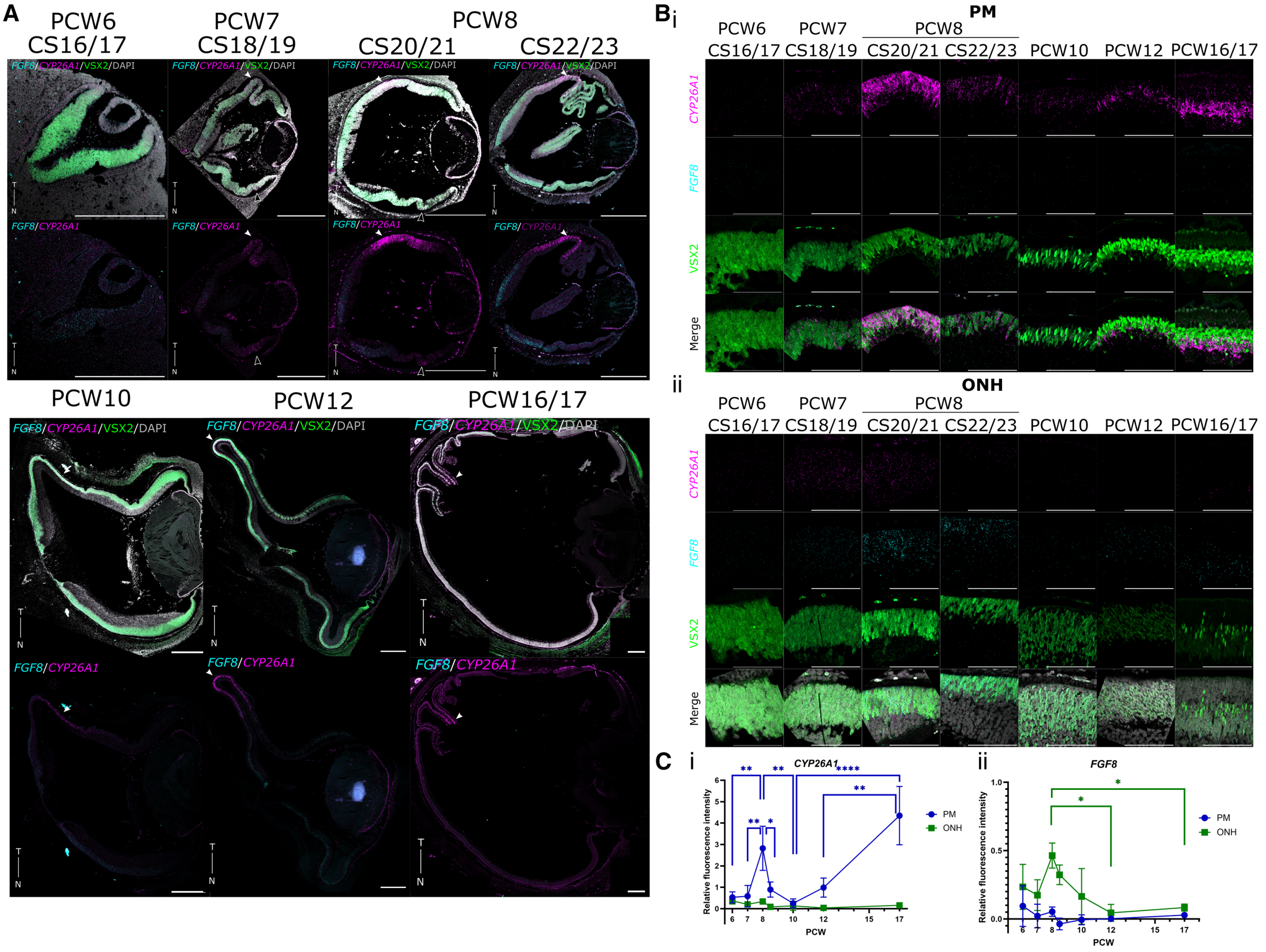

Figure 1. *CYP26A1* shows focal, biphasic expression within the PM of PCW6-17 human fetal retina, peaking at early PCW8 and PCW16/17

(A) Human fetal retina samples from PCW6-17 stained for *FGF8* (cyan) and RA catabolizing enzyme *CYP26A1* (magenta), showing *CYP26A1*$^+$ spot-like PM region colocalizing with RPC (retinal progenitor cell) marker, VSX2 (green) in the neuroblastic layer between PCW7 and PCW8 (CS18-23), and basal to the VSX2$^+$ layer at PCW12–17.

(B) Zoom-ins of the (i) PM and (ii) ONH.

(C) Quantification of *CYP26A1*/*FGF8* fluorescence intensity over PCW6–17 in PM/ONH, normalized to positive control (*POLR2A*/*PPIB*). One-way ANOVA yielded a statistically significant difference in *CYP26A1* expression in the PM between at least 2 time points ($F(6,15) = 13.96$, $p < 0.0001$), and Tukey's test found significant increase in mean fluorescence intensity between PCW7-early PCW8 (CS20-21) ($q(15) = 6.34$, $p = 0.006$), and a significant decrease between early PCW8 and late PCW8 (CS22-23) ($q(15) = 5.90$, $p = 0.01$). ANOVA found statistical differences in *CYP26A1* expression in the ONH ($F(6,14) = 3.39$, $p = 0.03$); however, no significance was identified by post-hoc tests. ANOVA yielded no significant differences in *FGF8* expression in the PM ($F(6,15) = 1.18$, $p = 0.40$) between any time points. Conversely, statistical differences were found in *FGF8* in the ONH ($F(6,15) = 4.06$, $p = 0.01$), with significantly higher *FGF8* at PCW8 compared to PCW12 ($q(15) = 5.45$, $p = 0.02$) and PCW17 ($q(15) = 4.95$, $p = 0.04$).

Images of probe labeling through entire CS20 retina, positive and negative controls, intensity measurements and all multiple comparison results for PM *CYP26A1* expression are shown in Figure S1. White arrows indicate PM, while white outlined arrow heads indicate low levels of *CYP26A1* expression visible at nasal edge of the retina. Sections counterstained with DAPI (greyscale). T, temporal; N, nasal; CS, Carnegie stage; PCW, post-conception weeks, PM, presumptive macula. *$p < 0.05$, **$p < 0.01$, and ***$p < 0.001$. Data shown as mean ± SD. Images taken at (A) 20×/(B) 40×, representative of $n \geq 2$ samples/time point. Scale bars: (A) 500 μm and (B) 100 μm.

with the highest expression seen at PCW8 (Figures 1Bii, 1Cii, and 3). Examining the whole retina, expression exhibited a strong ONH-to-periphery gradient (Figures 1A, 3A, and S1B), similar to the developing macaque (d40) (Krueger et al., 2024), mouse (E9.5–14.5) (Crossley and Martin, 1995),and chick eye (E2–E7) (Soukkarieh et al.,

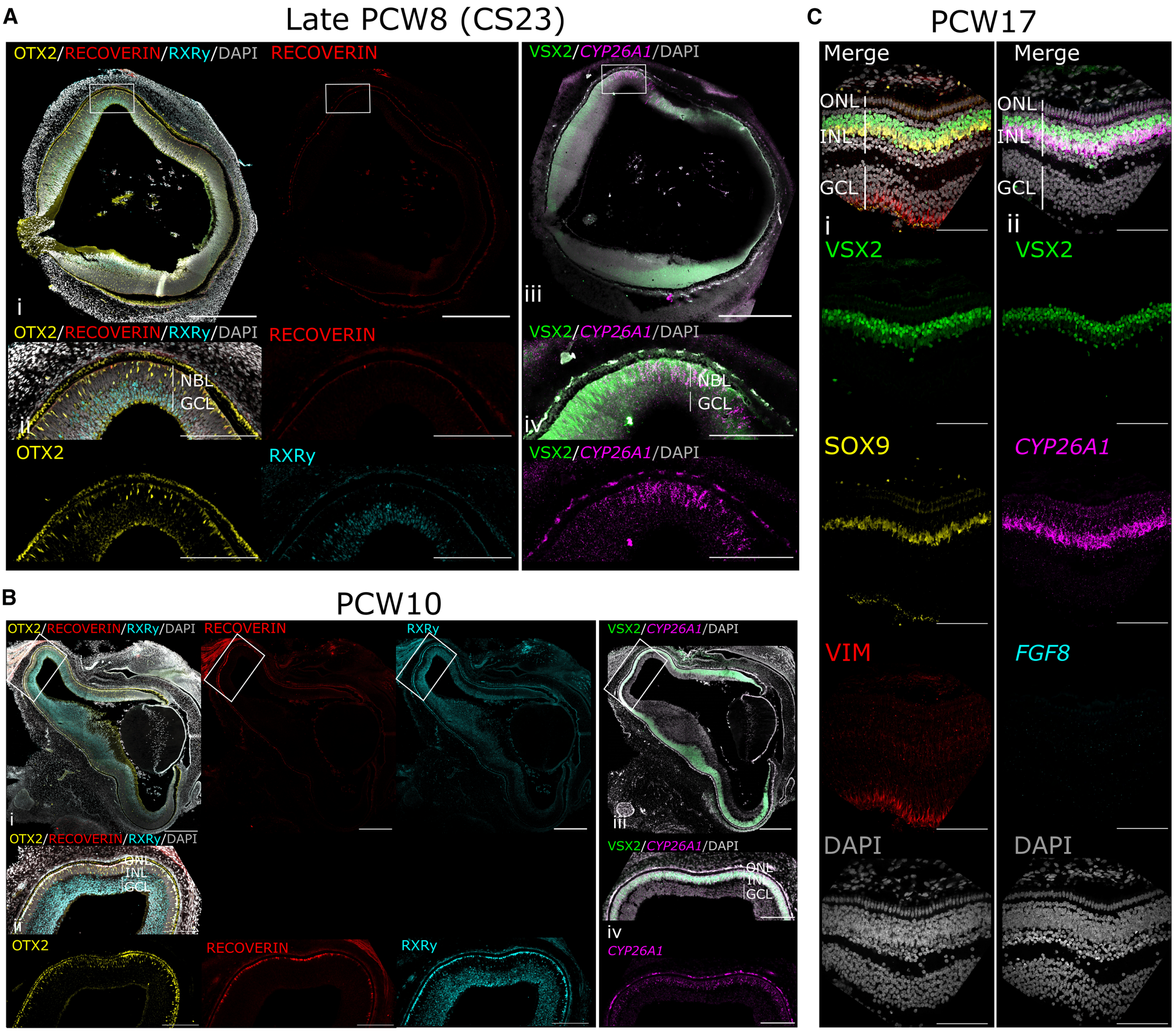

Figure 2. Cone progenitor markers are expressed in the NBL of late PCW8 PM, localized to the INL by PCW10 but do not co-express *CYP26A1*, while macular Müller Glia (MG) cells express *CYP26A1* in PCW17 human fetal retina

(A) At late PCW8 (CS23), OTX2 (yellow) is expressed in cone/bipolar-biased RPCs in the neuroblastic layer (NBL) throughout the retina, at highest density in the PM, while no RECOVERIN (red) or RXRγ$^+$ (cyan) cone precursor markers are visible.

(B) At PCW10, the PM region expresses a thick layer of OTX2$^+$ cells in the INL, and RXRγ$^+$/RECOVERIN$^+$ cone precursors localize to the apical single-cell thick ONL layer of the central retina, not present in the peripheral retina. *CYP26A1* expression is restricted to the INL, not expressed in the RECOVERIN$^+$/RXRγ$^+$/OTX2$^+$ cone precursors in the ONL.

(C) At PCW17, *CYP26A1* (magenta) is visible in the PM, localized to the VSX2$^+$ (green)/SOX9$^+$ (yellow) developing Müller glia layer, basal to the VSX2$^+$/SOX9$^-$ developing bipolar cell layer. Müller glia cytoskeletal marker vimentin (VIM, red) is clearly expressed, while no *FGF8* (cyan) is visible in the PM.

Stains were performed on consecutive sections from the same respective retina for Ai/iii, Bi/iii, and Ci/ii, and counterstained with DAPI (greyscale). CS, Carnegie Stage; PCW, post-conception weeks; NBL, neuroblastic layer; GCL, ganglion cell layer; ONL, outer nuclear layer; INL, inner nuclear layer. Images taken at (Ai/Bi) 20×/(Aii–Aiii/Bii and Biii/C) 40×. White box indicates magnified region of interest. Images representative of $n \geq 2$ samples. Scale bars: (A) 500 μm and (B and C) 100 μm.

2007), and from PCW12, *FGF8* labeling was also observed in the very peripheral retina, near the lens (Figure 1A). However, in contrast to the clearly demarcated, spot-like and spatiotemporally coincident expression of *Fgf8* and *Cyp26a1/c1* seen in the HAA of the chick, very little *FGF8* expression was observed within the *CYP26A1*-positive

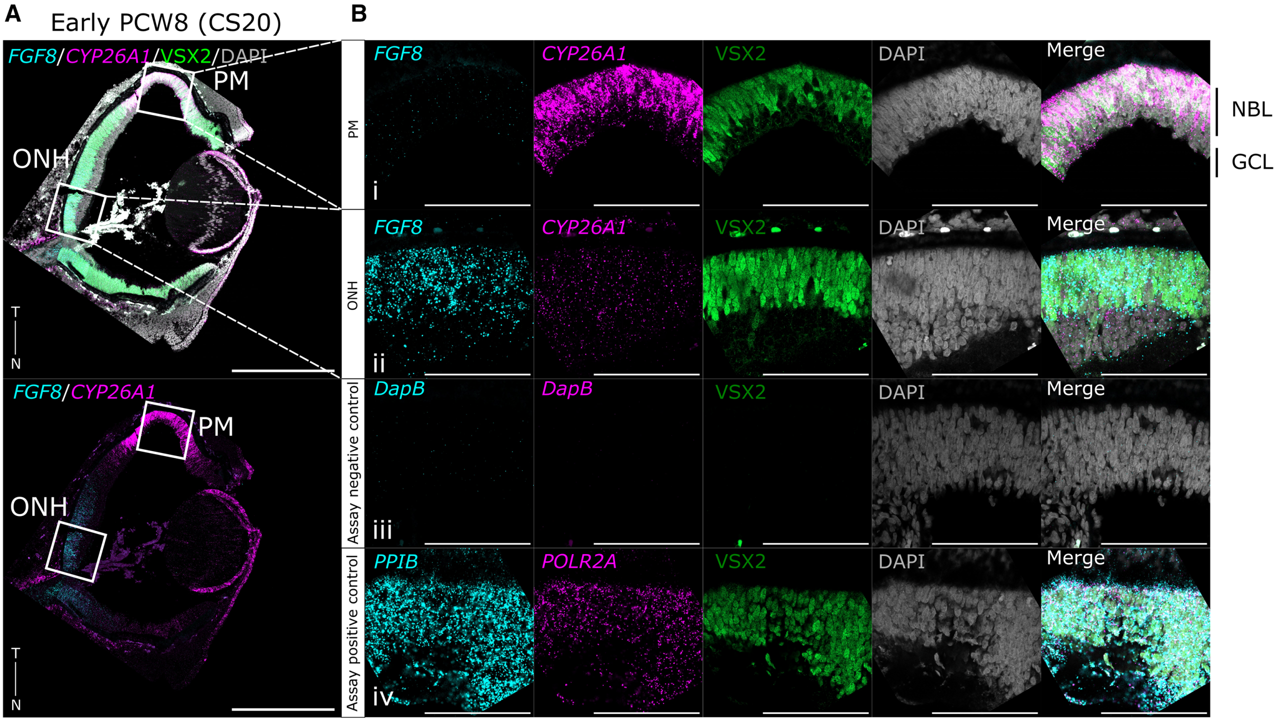

Figure 3. *CYP26A1* is highly expressed in the PM of early PCW8/CS20 human fetal retina, while *FGF8* is localized exclusively to the ONH

Early PCW8 (CS20) fetal retina stained *in situ* for *CYP26A1* (magenta) and *FGF8* (cyan), showing strong expression in the PM of *CYP26A1* colocalizing with RPC marker VSX2 (green), but no *FGF8*, which is visible in RPCs around the ONH. Assay negative control (*DapB*) and positive control (*PPIB* cyan, *POLR2A* magenta). Sections counterstained with DAPI (greyscale).

T, temporal; N, nasal; CS, Carnegie stage; PCW, post-conception weeks; PM, presumptive macula; ONH, optic nerve head. Images taken at 20× (B)/40× (A). Images representative of $n = 3$ samples. White box indicates magnified region of interest. Scale bars: (A) 500 μm and (B) 100 μm.

PM (Figures 1Bi, S1A, and S1B). While some limited signal was potentially discernible between PCW6 and PCW8 (Figure 1Cii), this was not specific to the PM (Figure S1B), and no quantitatively significant differences in expression within the PM were detected between any time points examined, including around periods of *CYP26A1* upregulation (PCW8/PCW17) (Figures 1Bi, 1Ci, S1A, and S1C).

The PM is located more peripherally in early development, relative to its central position in the mature retina, due to centre-to-periphery proliferation gradient

In the adult eye, the macula is located at the center of the retina, approximately 3.5–5.5 mm temporal to the ONH (Bringmann et al., 2018). At early developmental time points, however, the spot of *CYP26A1* expression identified as the PM was more peripheral than expected (Figure 1A), prompting us to examine growth and proliferation across regions of the retina throughout early development.

We measured the distance between the center of the region of *CYP26A1* expression (the PM) and (1) the peripheral edge of the retina; (2) the ONH (Figures 4Ai–4Aiii). The distance between PM-periphery increased from 300 to 9,000 μm between PCW7 and PCQ17, growing in a linear manner (R^2 = 0.97; Figures 1A and 4Aiv). The distance from PM-ONH also grew in a linear manner, from 400 to 6,000 μm (R^2 = 0.96; Figure 4Aiv), but the rate of increase was ∼2-fold lower (890 and 490 μm/week, respectively, $p > 0.001$). Notably, while the distance PM-periphery increases as a proportion of total retina length, from 20% at PCW7 to 45% at PCW17, PM-ONH remains constant at 30% (Figure 4Av).

To determine whether this relative positional shift of the PM was the result of variable proliferation across the retina, we examined regional proliferation using proliferative marker KI67 (Figure S2A). Consistent with the known center-to-periphery developmental gradient, and comparatively higher proliferation in the periphery at later stages of retinal development (Hendrickson, 2016; Hoshino et al., 2017), the proportion of Ki67$^+$ cells was ∼2-fold higher in the periphery compared to the central retina at both early and late PCW8 (early PCW8: peripheral 68% ± 11.6 vs.

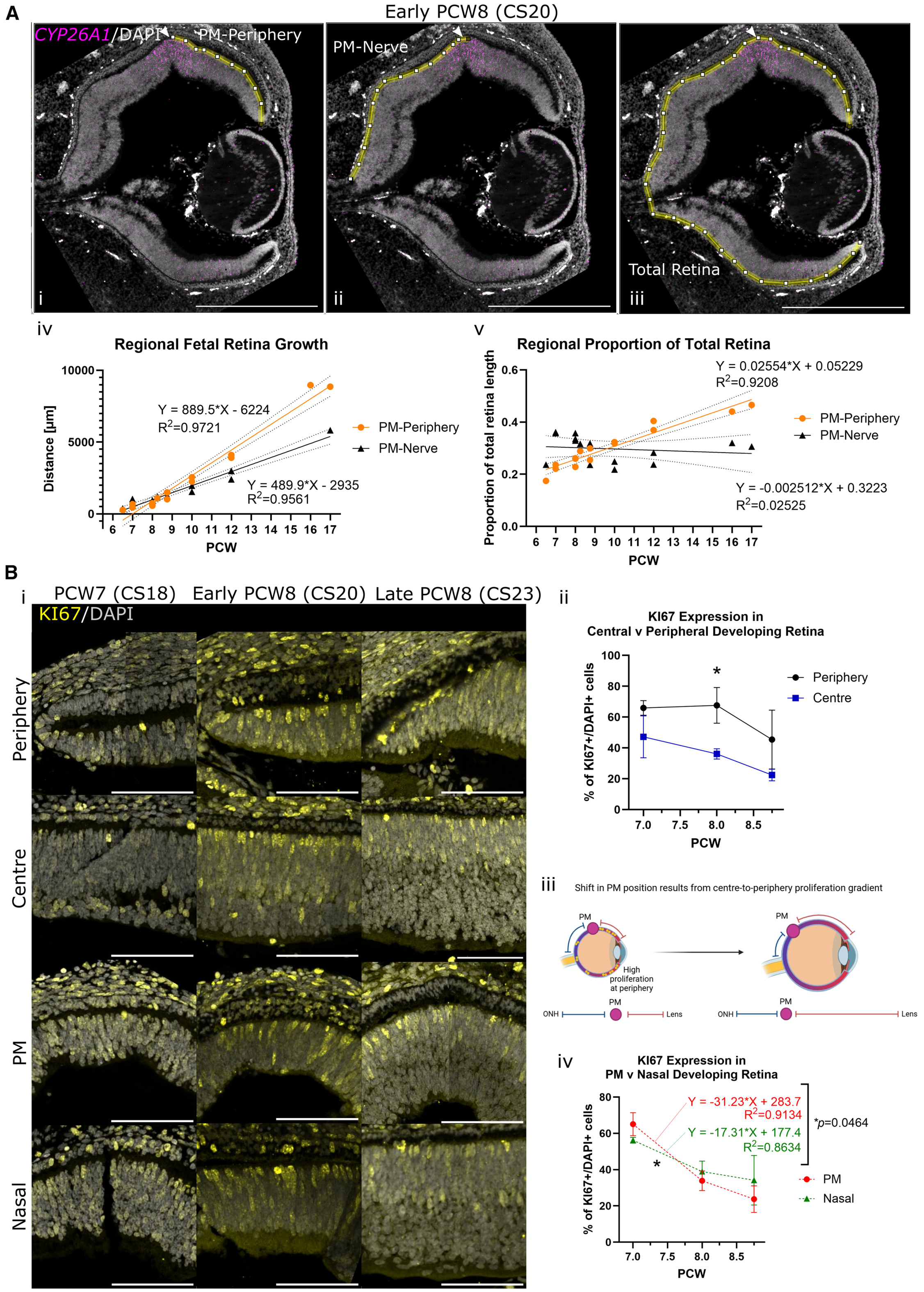

(legend on next page)

central 36% ± 3.4, late PCW8: peripheral 45% ± 19.1 vs. central 22% ± 3.7, respectively Figures 4Bi and 4Bii). The central-to-periphery proliferation gradient thereby explains the more peripheral location of the PM at early stages, compared to the adult eye (Figure 4Biii).

Reduction in proliferation marker KI67 in the PM coincides with *CYP26A1* expression at PCW8

We next examined proliferation within the PM during the first wave of *CYP26A1* expression, compared to a region in the nasal retina equidistant from the periphery, i.e., at the same position along on the central-peripheral developmental gradient (Figure S2A). As expected, both regions showed significant reductions in the proportion of proliferating cells between PCW7 and early PCW8 (PM: $t(4) = 6.5$, $p = 0.003$; nasal: $t(4) = 5.0$, $p = 0.007$; Figures 4Biv and S2B). However, linear regression analysis showed that the rate of reduction in the PM region was nearly twice that of the equivalent nasal region (−31.2%/week and −17.3%/week, respectively, $p = 0.046$) (Figures 4Biv and S2C). This indicates that the reduction in proliferation in the PM goes beyond that of the central-to-peripheral gradient; moreover, this rapid reduction in proliferation in the PM coincides with the first wave of *CYP26A1* expression.

hROs express components of RA synthesis and FGF8 pathways during early differentiation, demonstrating similarity to peripheral retina development

Our established 2D/3D differentiation protocol (Figure S3A) replicates the timings of human development, yielding hROs with ∼1:4 ratio of cones:rods, a proportion similar to the outer edges of the macula (Gonzalez-Cordero et al., 2017). Note that this protocol includes the addition of exogenous RA from d70, which is important for maintaining retinal structure in the later stages of maturation (Sanjurjo-Soriano et al., 2022) but by which time cone photoreceptor neurogenesis has already occurred (Figures S3B–S3E).

We examined the endogenous levels of RA and FGF8 signaling in our hROs. RA synthesizing enzymes, *ALDH1A1* and *ALDH1A3,* were both upregulated early in differentiation (d40/50), compared to undifferentiated human embryonic stem cells (hESCs) ($q(2) = 13.3$, $p = 0.02$; $q(2) = 23.2$, $p = 0.005$; Figures 5A and 5B). CRABP2 traffics cytoplasmic RA to the nucleus and is concomitantly upregulated with *all*-trans-RA, making it a proxy for circulating RA levels (Napoli, 2016). *CRABP2* expression increased throughout differentiation, particularly beyond d70, when exogenous RA is added ($q(2) = 7.3$, $p = 0.05$; Figure 5C). In contrast, *CYP26A1* expression was lower in d40 hROs, compared to undifferentiated hESCs ($q(2) = 15.4$, $p = 0.01$) and remained low until the addition of exogenous RA at d70, whereupon, unsurprisingly, it increased ($q(2) = 8.0$, $p = 0.04$) and remained raised until d150 (Figure 5D).

FGF8 was higher in d40 hROs compared to undifferentiated ESCs ($q(2) = 18.02$, $p = 0.008$), but expression decreased thereafter (Figure 5E), while *FGFR3* was upregulated between d40 and d90, (reaching significance at d50; $q(2) = 10.2$, $p = 0.02$), indicating FGF8 signaling is active over this period of hRO differentiation (Figure 5F). Expression of *FGFR1/2/4* remained low throughout (Figure 5F).

Early inhibition of RA by AGN193109 leads to smaller hROs and reduced proliferation

Given the striking biphasic expression of *CYP26A1* within the human PM, we sought to mimic these periods of reduced RA signaling in hROs to better understand their potential role(s) in macula formation. In human fetal retina, the first wave of *CYP26A1* peaks in early PCW8, prior to RXRγ and RECOVERIN protein expression (Figure 2A), which is equivalent to d40 in hROs (Figure S3E). We therefore attempted to recapitulate first

Figure 4. Higher proliferation in peripheral compared to central retina results in a temporally shifted *CYP26A1*$^+$ PM at early developmental stages PCW7–10

(A) (i–iii) The distance of the *CYP26A1*$^+$ (magenta) PM (white arrow) to the peripheral edge of the retina/ONH. (iv) PM-periphery distance increases at a significantly higher rate (>2-fold) between PCW7-17 compared with PM-ONH distance, $p < 0.001$. (v) The PM-periphery distance as a proportion of the whole retina increases from 20% to 45% over PCW7-17, while PM-ONH distance remains almost constant at 30% of the whole retina ($n = 14$, linear regression with 95% confidence internals plotted).

(B) (i) Representative images of proliferative marker KI67 (yellow) staining in PCW7-8 (CS18-23) human fetal retinas shows (ii) the proportion of DAPI$^+$ (gray) cells expressing KI67 was at ∼2-fold higher in the peripheral compared with central retina during PCW7-8 (PCW7: $t(2) = 3.3$, $p = 0.08$; early PCW8 (CS20): $t(2) = 6.4$, $p = 0.02$; late PCW8 (CS23): $t(2) = 2.5$, $p = 0.13$). (iii) Schematic showing centre-to-periphery proliferation gradient, and the peripheral location of the PM in early development gradually moving toward the center of the retina due to higher relative growth at the peripheral edge. (iv) Linear regression shows the decline in KI67$^+$/DAPI$^+$ cells in the PM region is significantly steeper than the nasal region between PCW7 and early PCW8, at which point *CYP26A1* is upregulated in the PM (PM: −32.2, nasal: −17.3, $p = 0.046$).

Region masking and full linear regression shown in Figure S2. Significance values determined by paired/unpaired t tests: $^*p < 0.05$ and $^{**}p < 0.01$. $n = 3$ samples/time point, data shown as mean ± SD. PCW, post-conception weeks; CS, Carnegie stage; PM, presumptive macula, ONH, optic nerve head. Images taken at 20×. Scale bars: (A) 500 μm and (B) 100 μm.

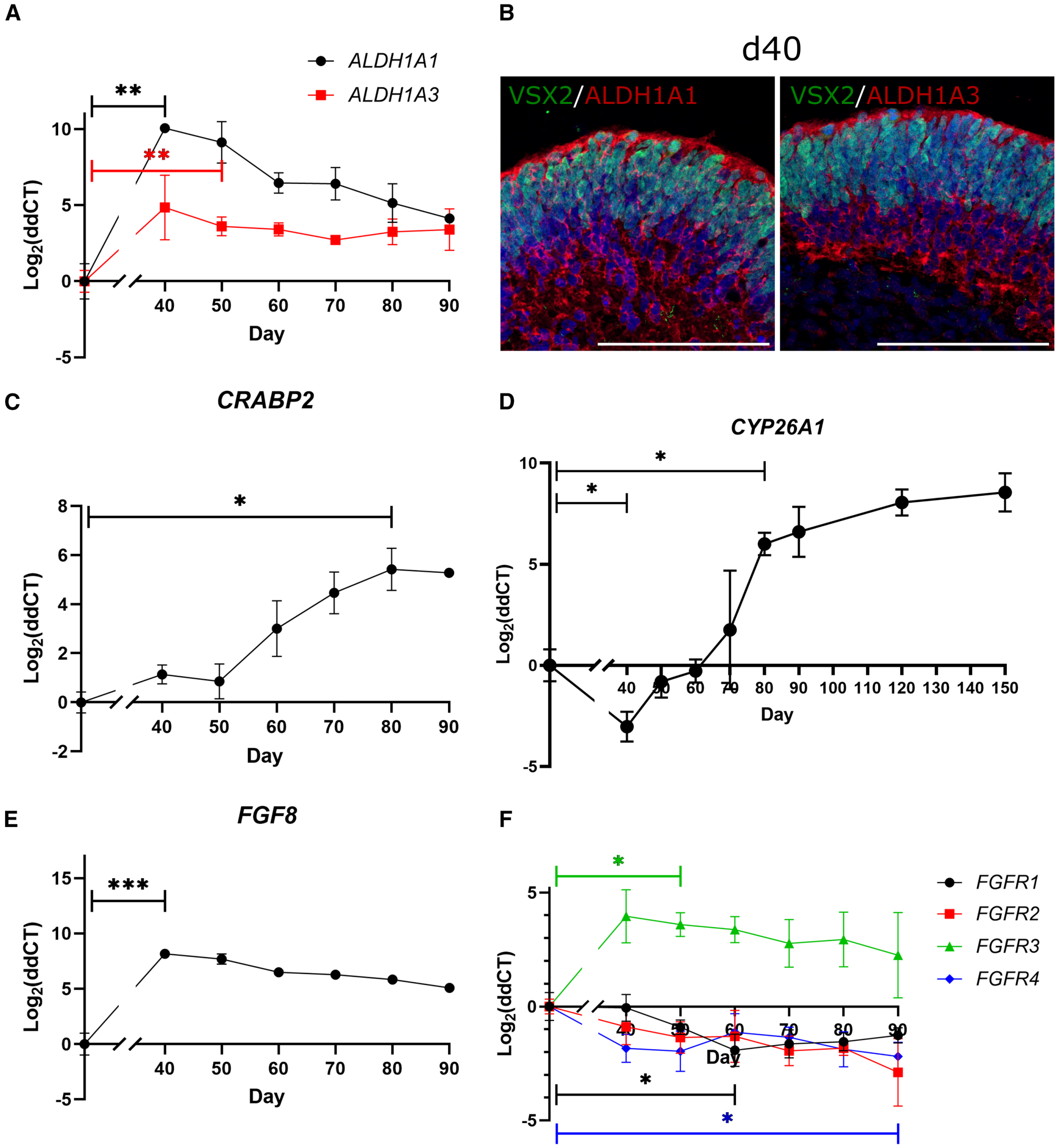

Figure 5. Differentiating human retinal organoids express retinoic acid and FGF8 pathway components

(A) Relative expression of RA synthesizing enzymes across hRO differentiation showing significant upregulation of *ALDH1A1* from d40 ($q(2) = 13.3$, $p = 0.02$), and *ALDH1A3* from d50 ($q(2) = 23.2$, $p = 0.005$).

(B) Immunostaining of ALDH1A1 and ALDH1A3 in d40 hROs showing expression throughout the organoid, co-stained with neural retinal progenitor cell marker VSX2, counterstained with DAPI (blue).

(C–F) Relative expression of mRNA for: (C) proxy for circulating RA levels *CRABP2,* with expression increasing from d40, but significantly upregulated at d80, following exogenous RA addition ($q(2) = 7.3$, $p = 0.05$); (D) retinoic acid catabolizing enzyme *CYP26A1*, initially downregulated compared to hESCs ($q(2) = 15.4$, $p = 0.01$), but upregulated at d80 following standard protocol addition of exogenous RA to culture medium from d70 ($q(2) = 8.0$, $p = 0.04$); (E) fibroblast growth factor 8 (*FGF8*), which has significantly increased expression at d40 ($q(2) = 18.02$, $p = 0.008$) and expression remains high, although slowly decreases up to d90; and (F) FGF receptors (*FGFR1/2/3/4*), which all showed slight downregulation compared to hESCs (*FGFR1* d60: $q(2) = 14.1$, $p = 0.01$; *FGFR4* d90: $q(2) = 8.2$, $p = 0.04$), except *FGFR3* that was significantly upregulated at d50 ($q(2) = 10.2$, $p = 0.02$), with expression remaining high, although slightly decreasing to d90.

(legend continued on next page)

wave of *CYP26A1*-induced RA catabolism by dosing hROs between d42 and d50 ("early") with pan-RAR antagonist AGN193109 (10 μM) (Figure S4A) (Schubert and Germain, 2023). Early dosing with AGN193109 significantly reduced expression of *CRABP2* ($t(2) = 5.77$, $p = 0.03$; Figure S5Ai) and *CYP26A1* ($t(3) = 7.91$, $p = 0.004$; Figure S5Ai), demonstrating that this dose effectively reduced levels of circulating RA. We also saw a concomitant significant increase in *FGF8* ($t(5) = 5.51$, $p = 0.003$; Figure S5Aii), indicating RA activity has been successfully inhibited, resulting in activation of downstream pathways (Cunningham and Duester, 2015).

Given the significant reduction in proliferation seen in the fetal PM during the first wave of *CYP26A1* expression (Figure 4Biv), we examined growth of hROs dosed with RA inhibitor AGN193109 to determine whether RA inhibition reduced proliferation. Indeed, RA inhibition between d42 and d50 significantly reduced growth by d60, compared with DMSO-treated controls ($t(32) = 5.18$, $p < 0.0001$), leading to significantly smaller hROs at d150, following cessation of proliferation at 15 weeks (Gonzalez-Cordero et al., 2017) ($t(16) = 2.66$, $p = 0.017$) (Figures 6A and S5B). Quantification of proliferative marker KI67 also showed a modest (~10%) but significant reduction in the proportion of $KI67^+$ cells in AGN193109-dosed hROs at d60, compared with DMSO-treated controls ($t(24) = 2.48$, $p = 0.02$) (Figure 6Bi). Conversely, there was no difference in the number of cells labeled with the apoptotic marker CASPASE-3 ($t(29) = 0.64$, $p = 0.5$) (Figure 6Bii).

Early pharmacological inhibition of RA signaling in hROs increases expression of photoreceptor markers, while addition of recombinant FGF8 has no effect

The aforementioned data suggest that, in keeping with previous studies that show RA regulates the balance of proliferation and neurogenesis (Todd et al., 2018), inhibition of RA between d42 and d50 promotes RPC cell cycle exit in hROs. We therefore examined whether this earlier cell cycle exit increased the adoption of early born cell fates. Indeed, we saw significantly increased expression of both pan-photoreceptor marker *RECOVERIN* ($t(5) = 2.56$, $p = 0.05$) and cone progenitor maker *OTX2* ($t(4) = 4.58$, $p = 0.01$) at d60 (Figure 6Ci). The proportion of $OTX2^+$ cells was also ~25% higher in early AGN193109-dosed hROs than controls ($t(28) = 2.4$, $p = 0.02$) (Figure 6D). No significant changes were observed in the expression of RPC marker *VSX2* (Figures 6Ci, 1D, and S5Di), cone progenitor markers *RXRγ*/*THRβ* (Figures 6Ci and S5Dii) or RGC marker *RBPMS* and RGC/bipolar/amacrine marker *ISLET1* (Figures S5Ci, S5Cii, and S5Diii) by d60. We also tested whether FGF8 signaling alters cone fate, as observed in chick, through addition of recombinant (r)FGF8 (100 ng/mL) between d42 and d50 (Figures S4B and S4C) but saw no changes in RPC or photoreceptor marker expression, compared with controls (Figure 6Cii).

Late RA inhibition results in decreased *S-OPSIN* and increased *M/L-OPSIN*, indicating a role in cone subtype specification

The aforementioned data indicate that the early wave of *CYP26A1* acts to promote cell cycle exit and the acquisition of a cone progenitor fate. We next sought to additionally mimic the second, later wave of *CYP26A1* expression. The "early+late" AGN193109 dosing protocol composed of the "early" pulse at d42–d50, as aforementioned, and two later pulses, the first at d70–d80, when $RXRY^+$/$OTX2^+$/$RECOVERIN^+$ hRO cone precursors have already been specified (Figure S3E), equivalent to the start of the late *CYP26A1* expression wave at PCW12 (Figures 1 and 2B), and the second at d120–d130, when hRO cones have matured and express *ARR3*, and rods express *RHODOPSIN* (Figures S3F–S3H), equivalent to high *CYP26A1* expression at PCW17 (Figures 1 and 2C). Note that we used two pulses, rather than sustained application, of AGN193109 in the later phase, as continuous removal of RA between d65 and d120 has previously been shown to cause loss of stratification in hROs (Sanjurjo-Soriano et al., 2022).

No change in pan-cone marker *ARRESTIN-3* or rod marker *RHODOPSIN* gene/protein expression was observed at d150 with either early-only or early+late dosing (Figures 7Ai, 7Aii, 7Bi, and 7Bii), indicating the overall proportions of cones and rods were not affected. However, those receiving early+late dosing showed a significant 2-fold reduction of *S-OPSIN* ($t(5) = 6.68$, $p = 0.001$) and a concurrent, significant ~4-fold increase in *L-OPSIN* ($t(5) = 3.27$, $p = 0.02$) and ~2-fold increase in *M-OPSIN* ($t(5) = 1.53$, $p = 0.04$), compared to DMSO controls (Figures 7Aiii–7Av). Conversely, hROs receiving early-only dosing showed no changes in cone subtype marker expression at d150 (Figures 7Aiii–7Avi). Immunostaining analysis revealed that early+late dosing with AGN193109 led to a significant reduction in the proportion of $ARR3^+$ cones expressing S-OPSIN protein ($t(11) = 3.14$, $p = 0.01$) (Figures 7Biii and

Further hRO marker expression shown in Figure S3. qPCR data shown as log_2 fold change of CT values relative to d0 hESCs ($n = 5$ pooled hROs/sample, $N = 3$). Significance values determined by one-way repeated measures ANOVA followed by Dunnett's test against control group, adjusting for multiple comparisons: *$p < 0.05$, **$p < 0.01$, and ***$p < 0.001$, data shown as mean ± SD. Staining images representative of $n = 3$ hROs from $N = 1$ batch. Scale bars: 100 μm.

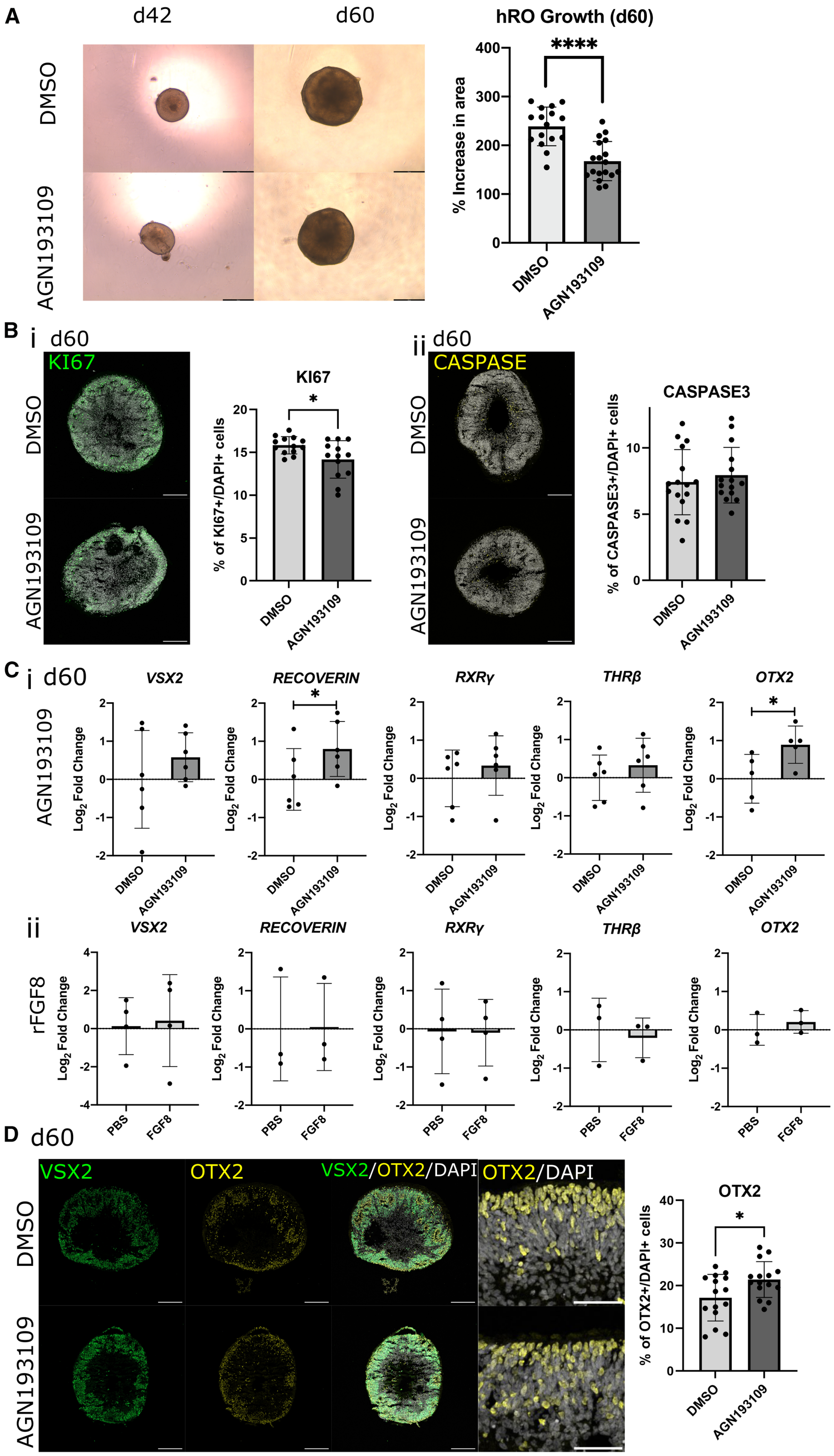

(legend on next page)

7Bv), although we did not observe a change in the proportion of M/L-OPSIN+/ARR3+ cones (Figures 7Biv and 7Bvi). These results indicate that inhibition of RA signaling later in cone development alters cone subtype specification.

DISCUSSION

Analysis of early-stage human macular development has been very limited to date, in particular prior to PCW8, with small sample sizes due to scarcity. The earliest previous *in situ* spatial analysis of *CYP26A1* expression in human fetal retina was late PCW8 (CS22-23), which showed expression in the PM at CS22 but none at CS23 (Silva and Cepko, 2017). Here, we provide the first spatiotemporal developmental timeline of *CYP26A1* expression in the developing human macula, revealing a striking biphasic pattern of expression. Importantly, we show that the first wave of *CYP26A1* expression is initiated in the RPCs of the PM at PCW7, prior to cone genesis, and is already reducing by late PCW8, while the second wave is evident from PCW12 and is specific to the MG within the INL of the PM. This second wave in the MG layer is in line with transcriptomic studies on adult human retina (Peng et al., 2019; Cowan et al., 2020), as well as a recently published single-nuclei gene expression data of PCW8–23 fetal retina, which showed *CYP26A1* was highly enriched in PCW17 macula cells (Zuo et al., 2024). Our findings are also consistent with a recent report of *CYP26A1* expression in rhesus macaque development (Krueger et al., 2024), which presented strong, biphasic *CYP26A1* expression in the PM region (Hendrickson and Zhang, 2019). In both human and macaque, *CYP26A1* was only expressed in RPCs during early macular formation, with no *CYP26A1* mRNA observed in the OTX2+/RXRγ+/RECOVERIN+ cone precursor cells of the ONL, indicating that RA inhibition may play a role in progenitor cell differentiation.

In contrast to HAA formation in the chick, where there is a striking spatiotemporal pattern of *Fgf8* expression in the HAA, we saw no indications of focal *FGF8* expression specific to the *CYP26A1*+ PM. Instead, *FGF8* expression remained low and exhibited no significant changes in expression in the human PM throughout PCW6–17 of development. Specifically, *FGF8* was not upregulated following *CYP26A1* expression in the PM at PCW8, in contrast to the chick HAA (da Silva and Cepko, 2017), suggesting additional/different regulatory mechanisms downstream of *CYP26A1* and low RA in the human macula. These findings are consistent with recent human fetal single-nuclei gene expression data, which found *FGF8* was enriched in peripheral, but not macula, RPCs (Zuo et al., 2024), and RNAscope analysis of rhesus macaque development, where *FGF8* was not enriched in the developing macula region at any time point examined (Krueger et al., 2024). Moreover, we found addition of rFGF8 to hRO cultures had no impact on photoreceptor marker expression. These suggest that *FGF8* is unlikely to play a significant role in human macula formation or macula cone differentiation. They also indicate a degree of divergence in the molecular mechanisms underlying high-acuity region development in different species and particularly warrant further investigation into the pathways downstream of *CYP26A1* in the human retina.

RPC differentiation and maturation occurs earlier in the macula than the rest of the retina, shown previously by primate and human studies (La Vail et al., 1991; Hendrickson, 1992; Provis and Hendrickson, 2008; Lu et al., 2020; Sridhar et al., 2020). While peripheral RPCs have been

Figure 6. Dosing early hROs with RA receptor inhibitor AGN193109 results in reduced proliferation and increased cone precursor marker OTX2 expression

(A) Following dosing between d42 and d50 ("early"), the % change in area from d42 to d60 was found to be significantly lower in AGN193109 dosed hROs compared with DMSO dosed controls, demonstrating reduced growth in RA inhibited hROs ($t(32) = 5.18$, $p < 0.0001$).

(B) Immunostaining for (i) apoptotic marker CASPASE-3 (yellow) at d60 showed no significant increase in cell death between AGN193109 dosed and control hROs ($t(29) = 0.64$, $p = 0.5$); (ii) proliferative marker KI67 (green) showed a significant reduction in cell proliferation in RA inhibited hROs ($t(24) = 2.48$, $p = 0.02$).

(C) (i) Dosing with AGN193109 led to significant increases in photoreceptor marker *RECOVERIN* ($t(5) = 2.56$, $p < 0.05$) and cone progenitor marker *OTX2* ($t(4) = 4.58$, $p = 0.01$), while early cone precursor markers *RXRγ* and *THRβ* trended toward increased expression ($t(5) = 1.45$, $p = 0.2$)/($t(5) = 1.69$, $p = 0.15$), with no increase in retinal progenitor cell (RPC) marker *VSX2* ($t(5) = 1.39$, $p = 0.2$). (ii) Dosing with rFGF8 had no effect on any marker expression tested (*RECOVERIN* $t(2) = 0.22$, $p = 0.85$; *OTX2* $t(2) = 2.99$, $p = 0.10$; *RXRγ* $t(3) = 0.23$, $p = 0.83$; *THRβ* $t(2) = 1.01$, $p = 0.40$; *VSX2* $t(3) = 0.39$, $p = 0.72$).

(D) Immunostaining of AGN193109 and DMSO dosed control hROs at d60 showed a significant increase in proportion of cone progenitor marker OTX2+ (yellow)/DAPI+ (gray) cells in AGN193109 dosed hROs ($t(28) = 2.4$, $p = 0.02$).

Further marker expression following early dosing shown in Figure S5. RT-qPCR data shown as log2 fold change of CT values relative to DMSO-treated controls ($n = 5$ pooled hROs/sample, $N \geq 3$). Significance values determined by paired (qPCR)/unpaired (IHC) t tests: $*p < 0.05$ and $****p < 0.0001$, $n \geq 13$, data shown as mean ± SD. Brightfield/staining images representative of $n \geq 13$ hROs from $N = 2$ batches. Scale bars: 200 µm.

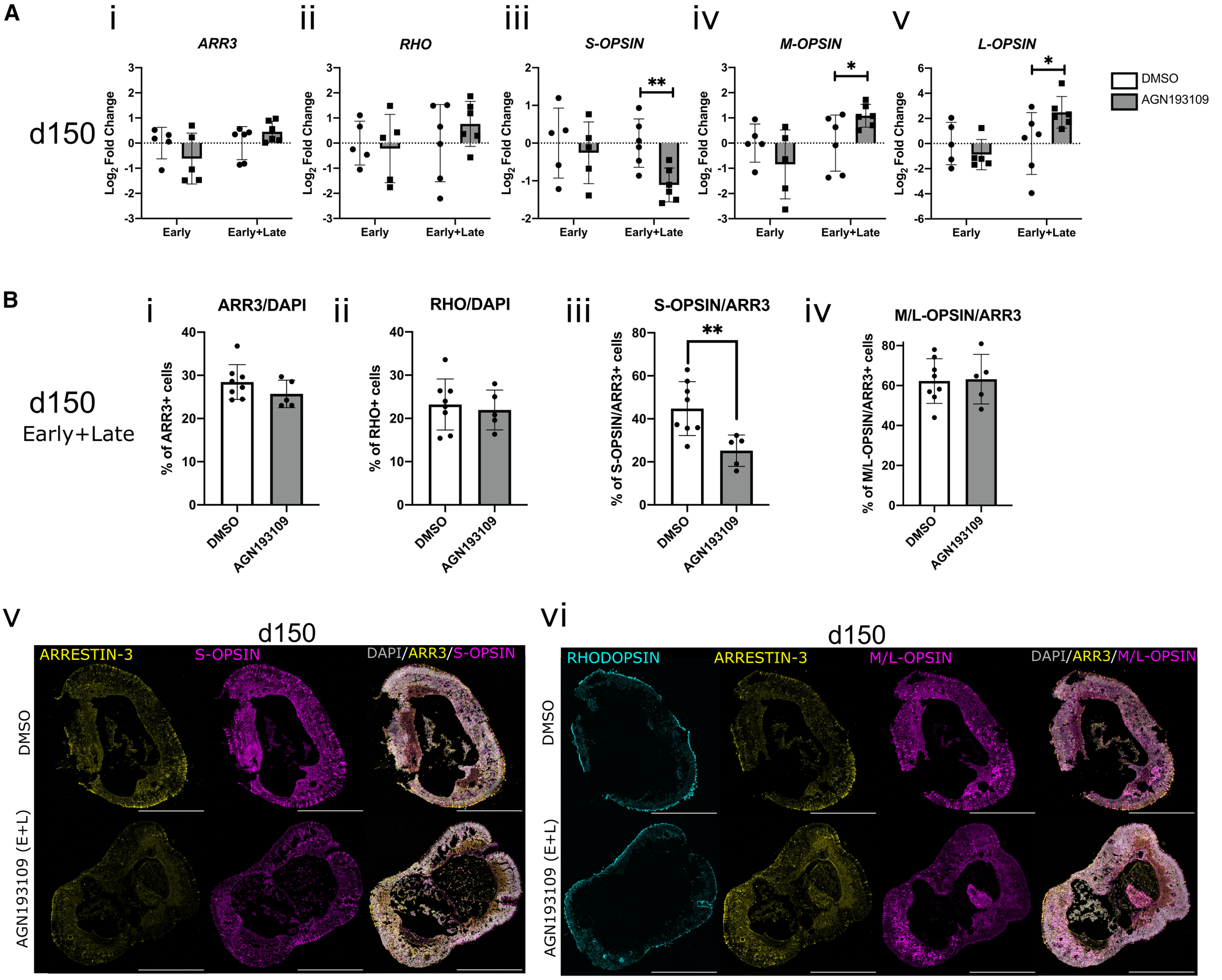

Figure 7. Additional late dosing of hROs with RA receptor inhibitor AGN193109 results in increased M/L-OPSIN and reduced S-OPSIN expression, indicating shift in cone subtype specification toward fovea-like composition

(A) Graphs show the effects of dosing with RA receptor inhibitor AGN193109 early in differentiation ("early"; d42–d50), or "early+late" (d42–d50 and d70–d80/d120–d130) on photoreceptor mRNA expression at d150. Early-only dosing had no effect on any marker expression tested: (i) *ARRESTIN*-3 $t(4) = 1.80$, $p = 0.15$; (ii) *RHODOPSIN* $t(4) = 0.63$, $p = 0.56$; (iii) *S-OPSIN* ($t(4) = 1.06$, $p = 0.35$); (iv) *M-OPSIN* $t(4) = 2.3$, $p = 0.08$); and (v) *L-OPSIN*, ($W = 9$, $n = 5$, $p = 0.31$). Early+late dosing lead to (i) no change in *ARRESTIN*-3, ($W = -15$, $n = 6$, $p = 0.16$); (ii) or *RHODOPSIN* ($t(5) = 2.48$ $p = 0.06$); (iii) a significant decrease in *S-OPSIN* ($t(5) = 6.68$, $p = 0.001$), (iv) a significant 1.8-fold increase in *M-OPSIN* ($t(5) = 2.82$, $p = 0.04$) and (v) a significant 3.5-fold increase in *L-OPSIN*, ($t(5) = 3.27$, $p = 0.02$).

(B) Addition of late pulses of AGN193109 (early+late) led to no change in proportion of $DAPI^{+}$ (gray) cells expressing (i) ARRESTIN-3 ($t(11) = 1.29$, $p = 0.22$) or (ii) RHODOPSIN ($t(11) = 0.41$, $p = 0.69$), (iii) but a significant decrease in the proportion of ARRESTIN-3^{+} cells expressing (iv) S-OPSIN ($t(11) = 3.14$, $p = 0.01$), (iv) although no change was observed in the proportion of ARRESTIN-3^{+} cells expressing M/L-OPSIN ($t(11) = 0.14$, $p = 0.89$). RT-qPCR data shown as $\log_2$ fold change of CT values relative to DMSO treated controls ($n = 3$ pooled hROs/sample, $N \geq 5$ samples/group). Significance values determined by paired (qPCR)/unpaired (IHC) t tests or Wilcoxon test: $*p < 0.05$ and $**p < 0.01$, data shown as mean ± SD. Staining data representative of $n > 5$ hROs from $N = 2$ batches. Scale bars: 500 μm.

shown to maintain their progenitor state for longer than those in the PM, and higher rates of proliferation are observed in the periphery from PCW8 (Hendrickson, 2016; Hoshino et al., 2017), earlier region-specific differences have not been studied due to lack of markers and access to tissue. Previously, bulk retina RNA-seq showed enrichment of proliferation-related genes between PCW7 and PCW10, and immunohistochemistry showed reduced KI67 expression in the central compared to peripheral retina at PCW8 (Hoshino et al., 2017). Developing this

further, we found that there is a marked reduction in proliferation specifically within the PM at early PCW8 (CS20-21), coincident with the first wave of *CYP26A1* expression. Given the known role of RA in maintaining the cell cycle (Kam et al., 2012), this first wave of *CYP26A1* appears to serve to promote early cell cycle exit within the PM.

We investigated *CYP26A1*'s roles in human macular development by inhibiting RA signaling in hROs via pharmacological blocking of RARs to mimic the low RA environment of the *CYP26A1*$^+$ PM. Given the coincident expression of *CYP26A1* and reduced proliferation in PCW7–8 PM, we asked whether early inhibition of RA encourages cell cycle exit and neurogenesis. Indeed, we observed significantly fewer proliferating cells and reduced growth in hROs exposed to early RAR inhibition, leading to significantly smaller hROs at both d60 and d150. This aligns with our previous data investigating the effects of exogenous RA on mouse ESC-derived retinal organoids (mROs), where the inverse occurs, and a short pulse of RA from d14 to d16 (cone precursor stage) led to an increase in proliferation and overall cell yield (Kruczek et al., 2017). In the current study, early-dosed hROs also displayed a significant increase in the number of OTX2$^+$ cells at d60, consistent with early cell cycle exit leading to differentiation of early-born cell types in the forming macula. However, this increase at d60 was not reflected in a concomitant increase at d150, presumably because supplementary pathways not active in the culture system, such as ONECUT1-mediated induction of thyroid signaling factors (Emerson et al., 2013; Eldred et al., 2018), are required for complete maturation into adult cones in hROs. Similarly, while the upregulation of *FGF8* in response to early RA-inhibition in hROs (Figure S5Aiii) is consistent with RA's known role in inhibiting *Fgf8* expression (Cunningham and Duester, 2015), it implies that in the human macula itself additional regulatory mechanisms may act to prevent *FGF8* upregulation in response to low RA concentration.

Additional pulses of RA inhibition, aimed at mimicking the second wave of *CYP26A1* expression, did not lead to a change in overall proportions of cones:rods in mature hROs, despite RA being known to promote rod production (Osakada et al., 2008), and removal of RA leading to more cone-rich organoids (Sanjurjo-Soriano et al., 2022). This may be because a more sustained period of low RA is required for a shift toward cone-rich hRO, since *in vivo* *CYP26A1* expression increases from PCW12 onwards and remains elevated for many weeks. As noted earlier, however, we did not try to replicate this since continuous removal of RA between d65 and d120 has previously been shown to cause loss of stratification in hROs(Sanjurjo-Soriano et al., 2022).

Cone subtypes are determined by two fate checkpoints: first, between S or M/L fate, and if the latter, between M- or L-OPSIN expression. The foveola at the center of the macula contains only M/L-cones while S-cones are present in the periphery of the retina. S-OPSIN-expressing cones are initially present in the early PM, and it is hypothesized that they later convert to M/L-cones, indicating plasticity of cone fate (Xiao and Hendrickson, 2000; Cornish et al., 2004). When RA signaling was inhibited early in differentiation, prior to cone neurogenesis, we observed no change in cone subtypes at d150. However, the addition of a later period of RA signaling inhibition led to a significant upregulation of *M/L-OPSIN* and concomitant downregulation of *S-OPSIN* gene expression and fewer S-OPSIN$^+$ cells. This reflects a recent BioRxiv report showing that *CYP26A1*-null hROs favor S-cone formation (Hussey et al., 2023). Interestingly, a similar effect was seen in *VAX2* knockout mice; normally *VAX2* restricts the expression of *CYP26A1* to a stripe in the central retina. In its absence, there is an expansion of M-cones into the usually S-cone dominated ventral retina (Alfano et al., 2011). Moreover, addition of exogenous RA to mROs after the period of cone precursor specification led to upregulation of S-opsin but no change in arrestin-3 expression, indicating that alterations in RA signaling later in development also influence S- vs. M/L-opsin cone subtype specification in mouse development (Kruczek et al., 2017).

RA has also recently been shown to regulate the M- vs. L-cone cell fate (Hadyniak et al., 2024). In our hands, while the addition of late pulses of RA inhibition in hROs led to an increase in both *M-* and *L-OPSIN* expression, we saw a greater impact on *L-OPSIN* expression, compared to *M-OPSIN* (~4-fold versus ~2-fold, increase respectively), indicating that lack of RA signaling more strongly favors L-cone fate. This broadly aligns with a recent report by Johnston and colleagues indicating that sustained application of RA to hROs during the same period of differentiation (d43–d130) of RA suppresses L-cone formation, while promoting M-cone fate (Hadyniak et al., 2024).

Together, these data show for the first time the spatiotemporal biphasic pattern of expression of *CYP26A1* within the PM across human macula development. Conversely, *FGF8* expression shows no such spatiotemporal changes in the PM, consistent with the macaque but in contrast to the chick HAA, suggesting a divergence of regulatory mechanisms in HAA formation. Based on our analysis of RA pathway manipulation in hRO models, we propose different roles for the two waves of CYP26A1-mediated RA inhibition in macular formation: the early wave (PCW7–8) stimulates RPCs to exit the cell cycle, encouraging cone generation, while the second, later wave (from PCW12) plays a role in the specification of cone subtypes.

This work bridges a gap in fundamental knowledge of the molecular mechanisms underlying human macular

development and highlights the importance (and challenges) of studying human development in both animal models and human-derived tissue. These results open new avenues for future research including resolving the molecular network downstream of *CYP26A1* and determining the drivers of *CYP26A1* expression in the human PM and how they are initiated. Elucidating these molecular mechanisms could provide strategies to encourage formation of more macular-like cellular structure in *in vitro* models to study macular development and disease, provide a preclinical model for drug testing to treat macular degeneration, and improve the efficacy of generating cells used for transplantation therapies that to restore visual function in patients.

METHODS

Human fetal retina sample collection
Human fetal retina samples were collected from the Human Developmental Biology Resource (HDBR) with research ethics approval and informed consent (Project, 200599; REC 18/LO/0822 and 18/NE/0290; IRAS: 244325 and 250012). Samples were stored in compliance with the Human Tissue Act 2004. Staging was performed by the HDBR according to defined criteria (https://hdbratlas.org/staging-criteria/carnegie-staging.html). A minimum of 2 biological replicates from independent samples were analyzed for each time point.

Differentiation of retinal organoids
Cells from the H9 ESC line were maintained and used to generate hROs according to a previously published protocol (Gonzalez-Cordero et al., 2017). Full protocol and reagents can be found in supplementary material. For dosing, 10 μM AGN193019 (Bioteche: 5758) or 100 ng/μL rFGF8 (Biotechne: 423-F8-025) were added to culture media every 2 days throughout the dosing windows.

RNAscope *in situ* hybridization
RNAscope multiplex fluorescence assay was performed using manufacturer's protocol for fixed frozen tissue with manual antigen retrieval. Sections were incubated with catalog probes Hs-CYP26A1-C1 (ACD Bio: 487741) and Hs-FGF8-C2 (ACD Bio: 415791-C2), counterstained with VSX2 and DAPI. Full protocol, reagents, and quantification can be found in supplementary material.

Immunohistochemistry
Immunohistochemistry (IHC) was performed using primary/secondary antibodies listed in Tables S1 and S2. Full protocol, reagents, and quantification can be found in supplementary material.

RT-qPCR gene expression analysis
Quantitative reverse-transcription PCR (RT-qPCR) was performed to analyze gene expression using primers listed in Table S3. Full protocol, reagents, and quantification can be found in supplementary material.

Fetal retina regional distance measurements
Regional length measurements were taken from confocal RNAscope images with fluorescent *CYP26A1* staining. The outer edge of the retina was manually traced using the segmented line drawing tool in ImageJ, from the center of *CYP26A1* expression (white arrow, Figure 4A) to the peripheral edge, or edge of optic stalk, and distance calculated using the measure function. Growth rates were analyzed using linear regression analysis in GraphPad Prism (v.10.1.2).

Statistical analyses
All data are presented as mean ± SD; *N* denotes number of independent experiments (i.e., differentiation batches) and *n* denotes number of images or hROs examined. Statistical testing was performed in GraphPad Prism (v.10.1.2). Tests used to analyze statistical significance are specified in methods/figure legends. Outliers were identified and excluded using the ROUT test ($Q = 10\%$). Shapiro-Wilk tests were used to assess normality and F-tests were used to confirm equal variances prior to applying parametric statistical tests.

RESOURCE AVAILABILITY

Lead contact
Further information and requests for resources/reagents should be directed to the corresponding authors.

Materials availability
This study did not generate new unique reagents.

Data and code availability
This paper did not generate original code. Additional data are available from the corresponding authors upon request. FAIR data management principles were followed throughout.

ACKNOWLEDGMENTS

This research was funded by Fight for Sight (5139/5140) and Medical Research Council UK (MR/T002735/2). Maja Wojtynska was funded by the Wellcome Trust as part of the Advanced Therapies for Regenerative Medicine Wellcome Trust PhD Program (218461/Z/19/Z). This work was made possible by the support, dedication, and teamwork of the Ocular Cell and Gene Therapy team, with particular thanks to Dr. E West, Dr. M Branch, Dr. M Khazim, B Ladino, J Kapadia, E Lanning, M Margari, M Tariq, C Mofidi, K Kumar, S Guilfoyle, and S van Heerden for help with stem cell maintenance cultures. We wish to thank Dr. M Riabiz (KCL) for providing independent expert statistical analysis advice. For

the purpose of open access, the authors have applied a Creative Commons Attribution (CC BY) license to any author accepted manuscript version arising. Graphical images were created in BioRender.

AUTHOR CONTRIBUTIONS

Conceptualization, P.H. and R.A.P.; data curation, P.H. and M.W.; formal analysis, P.H.; funding acquisition, R.A.P.; investigation, P.H. and R.A.P.; methodology, P.H. and R.A.P.; supervision, R.A.P.; visualization, P.H.; writing – original draft, P.H.; writing – review & editing, P.H., A.J.S., R.R.A., and R.A.P.

DECLARATION OF INTERESTS

The authors declare no competing interests.

SUPPLEMENTAL INFORMATION

Supplemental information can be found online at https://doi.org/10.1016/j.stemcr.2025.102647.

Received: November 19, 2024
Revised: August 26, 2025
Accepted: August 28, 2025
Published: September 25, 2025

REFERENCES

Alfano, G., Conte, I., Caramico, T., Avellino, R., Arnò, B., Pizzo, M.T., Tanimoto, N., Beck, S.C., Huber, G., Dollé, P., et al. (2011). Vax2 regulates retinoic acid distribution and cone opsin expression in the vertebrate eye. Development *138*, 261–271. https://doi.org/10.1242/DEV.051037.

Bringmann, A., Syrbe, S., Görner, K., Kacza, J., Francke, M., Wiedemann, P., and Reichenbach, A. (2018). The primate fovea: Structure, function and development. Prog. Retin. Eye Res. *66*, 49–84. https://doi.org/10.1016/j.preteyeres.2018.03.006.

Cornish, E.E., Xiao, M., Yang, Z., Provis, J.M., and Hendrickson, A.E. (2004). The role of opsin expression and apoptosis in determination of cone types in human retina. Exp. Eye Res. *78*, 1143–1154. https://doi.org/10.1016/J.EXER.2004.01.004.

Cowan, C.S., Renner, M., De Gennaro, M., Gross-Scherf, B., Goldblum, D., Hou, Y., Munz, M., Rodrigues, T.M., Krol, J., Szikra, T., et al. (2020). Cell Types of the Human Retina and Its Organoids at Single-Cell Resolution. Cell *182*, 1623–1640.e34. https://doi.org/10.1016/j.cell.2020.08.013.

Crossley, P.H., and Martin, G.R. (1995). The mouse Fgf8 gene encodes a family of polypeptides and is expressed in regions that direct outgrowth and patterning in the developing embryo. Development *121*, 439–451. https://doi.org/10.1242/DEV.121.2.439.

Cunningham, T.J., and Duester, G. (2015). Mechanisms of retinoic acid signalling and its roles in organ and limb development. Nat. Rev. Mol. Cell Biol. *16*, 110–123. https://doi.org/10.1038/nrm3932.

Curcio, C.A., Sloan, K.R., Kalina, R.E., and Hendrickson, A.E. (1990). Human photoreceptor topography. J. Comp. Neurol. *292*, 497–523. https://doi.org/10.1002/CNE.902920402.

Cvekl, A., and Wang, W.L. (2009). Retinoic acid signaling in mammalian eye development. Exp. Eye Res. *89*, 280–291. https://doi.org/10.1016/j.exer.2009.04.012.

Eldred, K.C., Hadyniak, S.E., Hussey, K.A., Brenerman, B., Zhang, P.W., Chamling, X., Sluch, V.M., Welsbie, D.S., Hattar, S., Taylor, J., et al. (2018). Thyroid hormone signaling specifies cone subtypes in human retinal organoids. Science *362*, eaau6348. https://doi.org/10.1126/SCIENCE.AAU6348.

Emerson, M.M., Surzenko, N., Goetz, J.J., Trimarchi, J., and Cepko, C.L. (2013). Otx2 and Onecut1 promote the fates of cone photoreceptors and horizontal cells and repress rod photoreceptors. Dev. Cell *26*, 59–72. https://doi.org/10.1016/J.DEVCEL.2013.06.005.

Gonzalez-Cordero, A., Kruczek, K., Naeem, A., Fernando, M., Kloc, M., Ribeiro, J., Goh, D., Duran, Y., Blackford, S.J.I., Abelleira-Hervas, L., et al. (2017). Recapitulation of Human Retinal Development from Human Pluripotent Stem Cells Generates Transplantable Populations of Cone Photoreceptors. Stem Cell Rep. *9*, 820–837. https://doi.org/10.1016/j.stemcr.2017.07.022.

Hadyniak, S.E., Hagen, J.F.D., Eldred, K.C., Brenerman, B., Hussey, K.A., McCoy, R.C., Sauria, M.E.G., Kuchenbecker, J.A., Reh, T., Glass, I., et al. (2024). Retinoic acid signaling regulates spatiotemporal specification of human green and red cones. PLoS Biol. *22*, e3002464. https://doi.org/10.1371/JOURNAL.PBIO.3002464.

Hendrickson, A. (1992). A morphological comparison of foveal development in man and monkey. Eye *6*, 136–144. https://doi.org/10.1038/eye.1992.29.

Hendrickson, A. (2016). Development of Retinal Layers in Prenatal Human Retina. Am. J. Ophthalmol. *161*, 29–35.e1. https://doi.org/10.1016/J.AJO.2015.09.023.

Hendrickson, A., and Zhang, C. (2019). Development of cone photoreceptors and their synapses in the human and monkey fovea. J. Comp. Neurol. *527*, 38–51. https://doi.org/10.1002/CNE.24170.

Hoshino, A., Ratnapriya, R., Brooks, M.J., Chaitankar, V., Wilken, M.S., Zhang, C., Starostik, M.R., Gieser, L., La Torre, A., Nishio, M., et al. (2017). Molecular Anatomy of the Developing Human Retina. Dev. Cell *43*, 763–779.e4. https://doi.org/10.1016/J.DEVCEL.2017.10.029.

Hussey, K.A., Eldred, K., Reh, T., and Johnston, R.J., Jr. (2023). Foveolar cone subtype patterning in human retinal organoids. Preprint at bioRxiv. https://doi.org/10.1101/2023.01.28.526051.

Kam, R.K.T., Deng, Y., Chen, Y., and Zhao, H. (2012). Retinoic acid synthesis and functions in early embryonic development. Cell Biosci. *2*, 11–14. https://doi.org/10.1186/2045-3701-2-11.

Kruczek, K., Gonzalez-Cordero, A., Goh, D., Naeem, A., Jonikas, M., Blackford, S.J.I., Kloc, M., Duran, Y., Georgiadis, A., Sampson, R.D., et al. (2017). Differentiation and Transplantation of Embryonic Stem Cell-Derived Cone Photoreceptors into a Mouse Model of End-Stage Retinal Degeneration. Stem Cell Rep. *8*, 1659–1674. https://doi.org/10.1016/j.stemcr.2017.04.030.

Krueger, M.R., Fishman-Williams, E., Simó, S., Tarantal, A.F., and La Torre, A. (2024). Expression patterns of CYP26A1, FGF8, CDKN1A, and NPVF in the developing rhesus monkey retina. Differentiation *135*, 100743. https://doi.org/10.1016/J.DIFF.2023.100743.

Lahne, M., Yoshimatsu, T., and MacDonald, R.B. (2023). Retinoic acid signaling in the developing zebrafish high acuity zone. Investig. Ophthalmol. Vis. Sci. *64*, 3630.

Lu, Y., Shiau, F., Yi, W., Lu, S., Wu, Q., Pearson, J.D., Kallman, A., Zhong, S., Hoang, T., Zuo, Z., et al. (2020). Single-Cell Analysis of Human Retina Identifies Evolutionarily Conserved and Species-Specific Mechanisms Controlling Development. Dev. Cell *53*, 473–491.e9. https://doi.org/10.1016/j.devcel.2020.04.009.

Mey, J., and Thanos, S. (2000). Development of the visual system of the chick: I. Cell differentiation and histogenesis. Brain Res. Rev. *32*, 343–379. https://doi.org/10.1016/S0165-0173(99)00022-3.

Napoli, J.L. (2016). Functions of Intracellular Retinoid Binding-Proteins. Subcell. Biochem. *81*, 21–76. https://doi.org/10.1007/978-94-024-0945-1_2.

Osakada, F., Ikeda, H., Mandai, M., Wataya, T., Watanabe, K., Yoshimura, N., Akaike, A., Sasai, Y., and Takahashi, M. (2008). Toward the generation of rod and cone photoreceptors from mouse, monkey and human embryonic stem cells. Nat. Biotechnol. *26*, 215–224. https://doi.org/10.1038/nbt1384.

Peng, Y.R., Shekhar, K., Yan, W., Herrmann, D., Sappington, A., Bryman, G.S., van Zyl, T., Do, M.T.H., Regev, A., and Sanes, J.R. (2019). Molecular Classification and Comparative Taxonomics of Foveal and Peripheral Cells in Primate Retina. Cell *176*, 1222–1237.e22. https://doi.org/10.1016/J.CELL.2019.01.004.

Provis, J.M., and Hendrickson, A.E. (2008). The Foveal Avascular Region of Developing Human Retina. Arch. Ophthalmol. *126*, 507–511. https://doi.org/10.1001/ARCHOPHT.126.4.507.

Sanjurjo-Soriano, C., Erkilic, N., Damodar, K., Boukhaddaoui, H., Diakatou, M., Garita-Hernandez, M., Mamaeva, D., Dubois, G., Jazouli, Z., Jimenez-Medina, C., et al. (2022). Retinoic acid delays initial photoreceptor differentiation and results in a highly structured mature retinal organoid. Stem Cell Res. Ther. *13*, 478. https://doi.org/10.1186/S13287-022-03146-X.

Schubert, M., and Germain, P. (2023). Retinoic Acid and Retinoid X Receptors. Cells *12*, 864. https://doi.org/10.3390/CELLS12060864.

da Silva, S., and Cepko, C.L. (2017). Fgf8 Expression and Degradation of Retinoic Acid Are Required for Patterning a High-Acuity Area in the Retina. Dev. Cell *42*, 68–81.e6. https://doi.org/10.1016/j.devcel.2017.05.024.

Soukkarieh, C., Agius, E., Soula, C., and Cochard, P. (2007). Pax2 regulates neuronal–glial cell fate choice in the embryonic optic nerve. Dev. Biol. *303*, 800–813. https://doi.org/10.1016/J.YDBIO.2006.11.016.

Sridhar, A., Hoshino, A., Finkbeiner, C.R., Chitsazan, A., Dai, L., Haugan, A.K., Eschenbacher, K.M., Jackson, D.L., Trapnell, C., Bermingham-McDonogh, O., et al. (2020). Single-Cell Transcriptomic Comparison of Human Fetal Retina, hPSC-Derived Retinal Organoids, and Long-Term Retinal Cultures. Cell Rep. *30*, 1644–1659.e4. https://doi.org/10.1016/j.celrep.2020.01.007.

Todd, L., Suarez, L., Quinn, C., and Fischer, A.J. (2018). Retinoic Acid-Signaling Regulates the Proliferative and Neurogenic Capacity of Müller Glia-Derived Progenitor Cells in the Avian Retina. Stem Cells *36*, 392–405. https://doi.org/10.1002/STEM.2742.

La Vail, M.M., Rapaport, D.H., and Rakic, P. (1991). Cytogenesis in the monkey retina. J. Comp. Neurol. *309*, 86–114. https://doi.org/10.1002/CNE.903090107.

Voigt, A.P., Mullin, N.K., Whitmore, S.S., DeLuca, A.P., Burnight, E.R., Liu, X., Tucker, B.A., Scheetz, T.E., Stone, E.M., and Mullins, R.F. (2021). Human photoreceptor cells from different macular subregions have distinct transcriptional profiles. Hum. Mol. Genet. *30*, 1543–1558. https://doi.org/10.1093/hmg/ddab140.

Xiao, M., and Hendrickson, A. (2000). Spatial and temporal expression of short, long/medium, or both opsins in human fetal cones. J. Comp. Neurol. *425*, 545–559.

Zuo, Z., Cheng, X., Ferdous, S., Shao, J., Li, J., Bao, Y., Li, J., Lu, J., Jacobo Lopez, A., Wohlschlegel, J., et al. (2024). Single cell dual-omic atlas of the human developing retina. Nat. Commun. *15*, 6792. https://doi.org/10.1038/s41467-024-50853-5.

Stem Cell Reports, Volume *20*

Supplemental Information

Human macula formation involves two waves of retinoic acid suppression via *CYP26A1* that modulate cell cycle exit and cone subtype specification

Philippa Harding, Maja Wojtynska, Alexander J. Smith, Robin R. Ali, and Rachael A. Pearson

Supplementary Figures

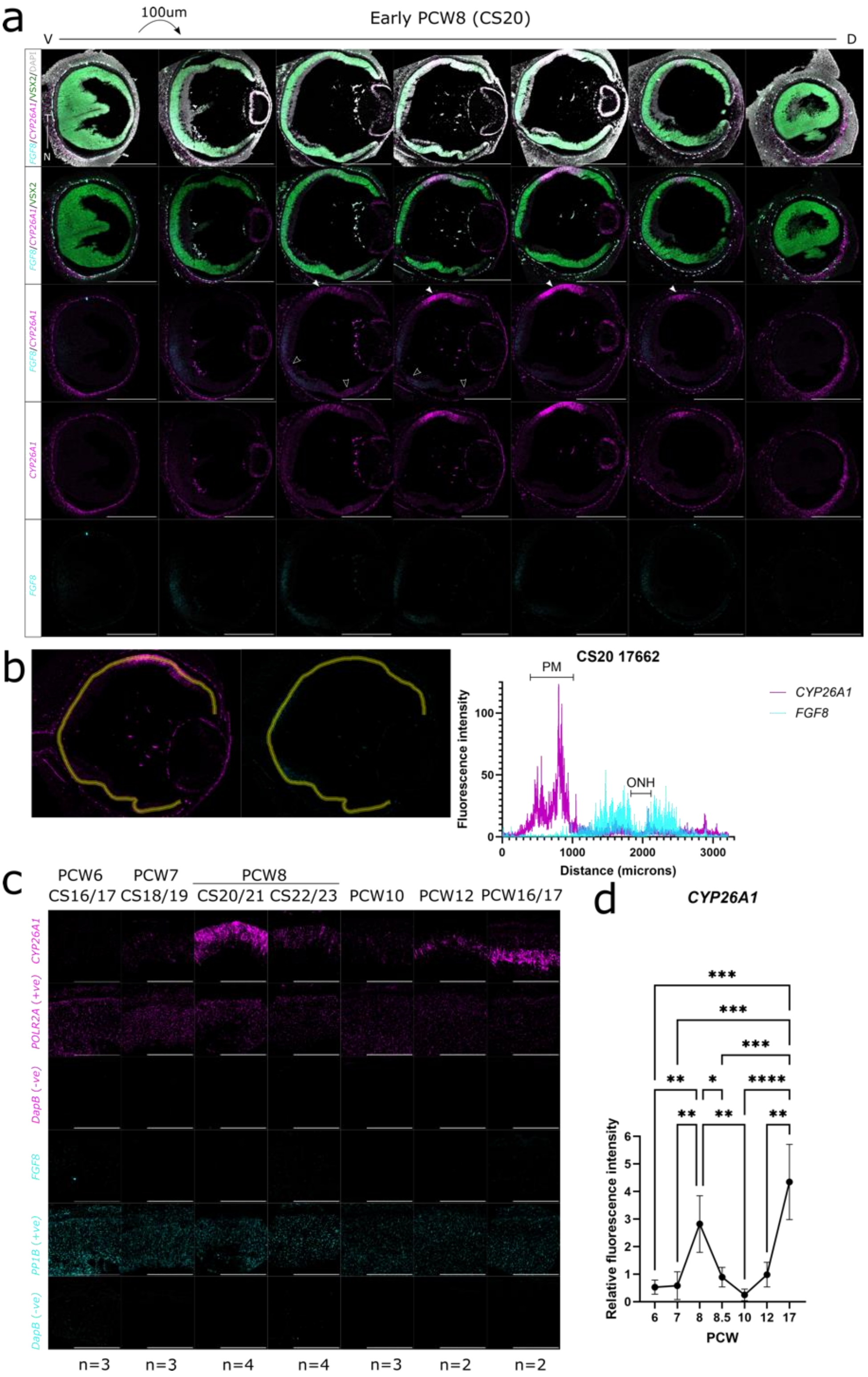

Fig.S1. *CYP26A1* expression is localized to a single spot-like region at the presumptive macula (PM), with expression peaks at early PCW8 (CS20-21) and from PCW12 onwards, while *FGF8* is exclusively expressed around the optic nerve head, related to Fig.1. (a) Representative images of serial sections through a CS20 human retina, taken at 100µm intervals and stained for the RA catabolizing enzyme, *CYP26A1* (magenta), and growth factor *FGF8* (cyan) mRNA using RNAscope™. Imaging shows expression of *CYP26A1* in the PM with no overlapping FGF8 expression, which is instead expressed around the optic nerve head. (b) Quantification of *CYP26A1*/*FGF8* fluorescence intensity across a single retina at CS20, showing *FGF8* is expressed in the retinal regions around optic nerve head, while *CYP26A1* is specifically expressed in the presumptive macula, where there is no distinct, overlapping *FGF8* expression. (c) Representative high-magnification images of the *CYP26A1*+ PM regions at all timepoints, including assay positive (*PPIB* cyan, *POLR2A*, magenta) and negative (*DapB*) controls showing consistent background/housekeeping expression across biological samples. (d) Quantification of *CYP26A1* normalized fluorescence intensity in the PM including all statistically significant values based on Tukey's multiple comparisons tests following one-way ANOVA. **p* value<0.05, ***p* value<0.01, *** *p* value<0.001, **** *p* value<0.0001. Data are represented as mean ±SD. White arrows indicate PM, while white outlined arrow heads indicate low levels of *CYP26A1* expression visible around optic nerve head (ONH) and at nasal edge of the retina. Sections were co-stained with RPC marker, VSX2 (green) and counterstained with nuclear marker, DAPI (greyscale). S – Superior; I – Inferior; T – Temporal; N – Nasal; CS – Carnegie Stage; PCW – Post-conception weeks, PM – Presumptive macula. Images taken at 20x magnification. Images are representative of n=3 CS20/21 samples. Scale bars - 500µm.

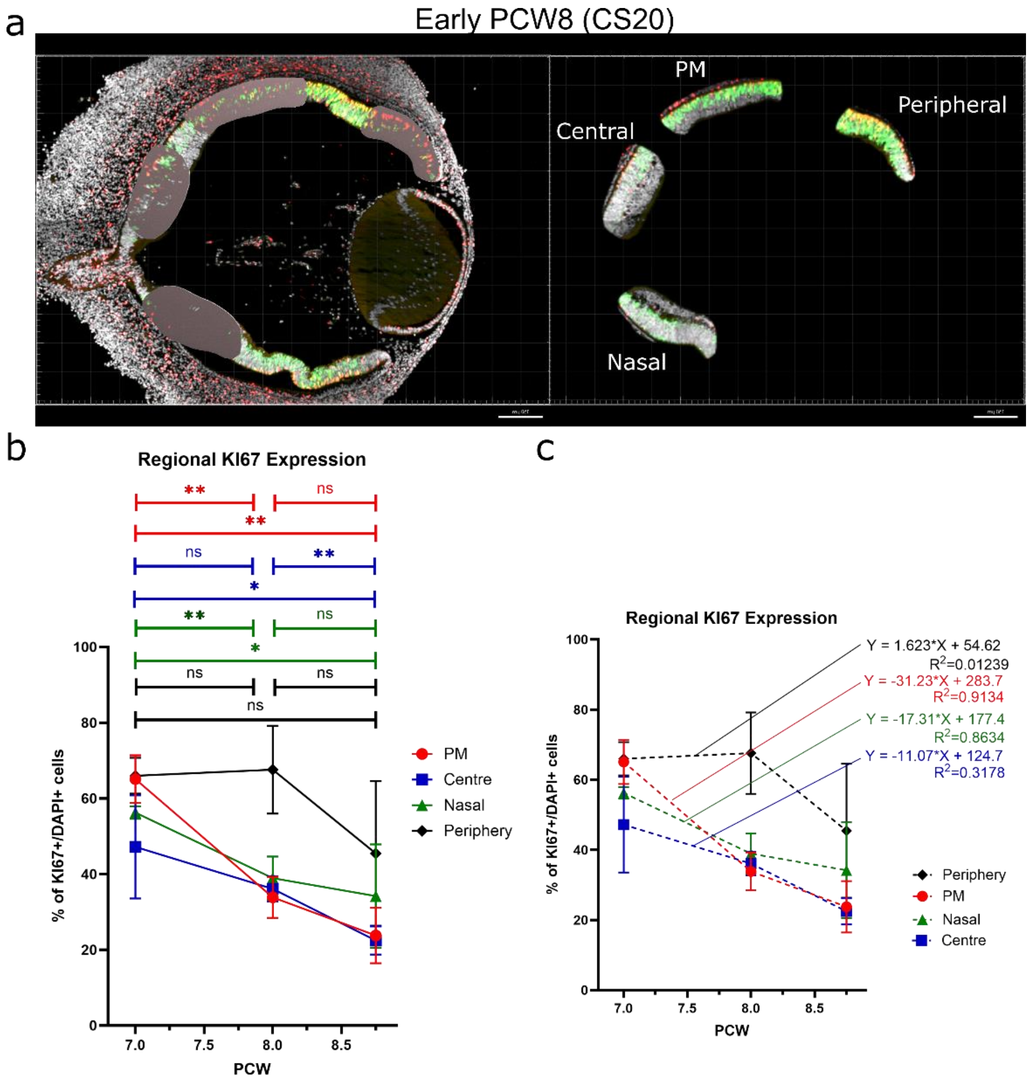
a
Early PCW8 (CS20)
PM
Peripheral
Central
Nasal
b
Regional KI67 Expression
**
ns
**
ns
**
*
**
ns
*
ns
ns
ns
% of KI67+/DAPI+ cells
PCW
PM
Centre
Nasal
Periphery
c
Regional KI67 Expression
Y = 1.623*X + 54.62
R2=0.01239
Y = -31.23*X + 283.7
R2=0.9134
Y = -17.31*X + 177.4
R2=0.8634
Y = -11.07*X + 124.7
R2=0.3178
% of KI67+/DAPI+ cells
PCW
Periphery
PM
Nasal
Centre

24

Fig.S2. Proliferation in human developing retinal regions (CS18-23), related to Fig.4. (a), Masking of PM (based on *CYP26A1*+ staining), central (adjacent to the optic nerve), nasal (equivalent distance as PM to periphery on the central-peripheral gradient on the nasal side) and peripheral regions in Imaris 10.0. (b) Proportion of KI67+/DAPI+ cells in all analyzed developing retinal regions from CS18-23. The proportion of proliferating DAPI+ cells decreases significantly in both the PM and the equivalent nasal region on the center to peripheral gradient by early PCW8 (PM: $t(4)=6.50$, $p=0.003$; nasal: $t(4)=5.03$, $p=0.007$) as well as between PCW7 and late PCW8 (PM: $t(4)=7.4$, $p=0.002$; nasal: $t(4)=2.8$, $p=0.05$). At CS18, the PM has a similar proportion of KI67+/DAPI+ cells to the peripheral retina (mean of 65/66% respectively, $t(2)=0.33$, $p=0.78$)), while at CS20/23, the PM had similar proportion of KI67+/DAPI+ cells to the central retina (CS20: means of 34/36%, $t(2)=1.15$, $p=0.37$); CS23: means of 24/22%, $t(2)=0.38$, $p=0.74$) and significant/nearing significant difference in proliferating cells compared with nasal/peripheral retina (CS20: $t(2)=5.26$, $p=0.03$/$t(2)=5.92$, $p=0.02$; CS23: $t(2)=2.47$, $p=0.13$/$t(2)=2.76$, $p=0.11$ respectively. (c) Linear regression of proportion of KI67+/DAPI+ cells in different retinal regions between CS18 and CS20 shows that the PM region has a steeper decline in KI67+ cells than any other retinal region (PM: -15.62, central: -5.5, nasal: -8.7, peripheral: 0.8, p=0.01). PM – Presumptive Macula; PCW – Post-conception Weeks. n=3/timepoint, data are represented as mean ±SD.

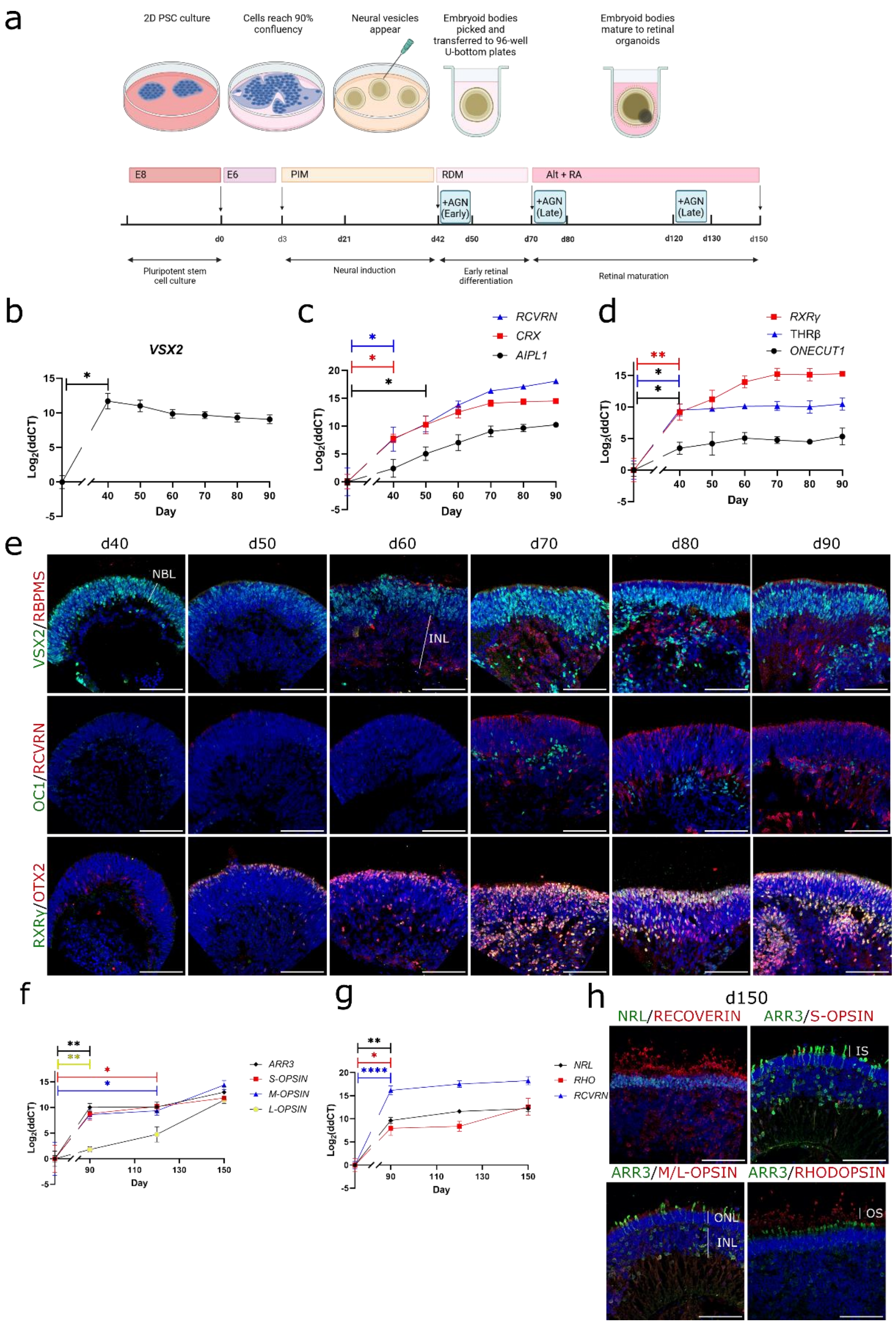
a
2D PSC culture
Cells reach 90% confluency
Neural vesicles appear
Embryoid bodies picked and transferred to 96-well U-bottom plates
Embryoid bodies mature to retinal organoids
E8
E6
PIM
RDM
Alt + RA
+AGN (Early)
+AGN (Late)
+AGN (Late)
d0
d3
d21
d42
d50
d70
d80
d120
d130
d150
Pluripotent stem cell culture
Neural induction
Early retinal differentiation
Retinal maturation
b
VSX2
Log2(ddCT)
Day
c
RCVRN
CRX
AIPL1
d
RXRγ
THRβ
ONECUT1
e
d40
d50
d60
d70
d80
d90
VSX2/RBPMS
NBL
INL
OC1/RCVRN
RXRγ/OTX2
f
ARR3
S-OPSIN
M-OPSIN
L-OPSIN
g
NRL
RHO
RCVRN
h
d150
NRL/RECOVERIN
ARR3/S-OPSIN
IS
ARR3/M/L-OPSIN
ARR3/RHODOPSIN
ONL
INL
OS

Fig.S3. Temporal and spatial photoreceptor development in human retinal organoids, related to Fig.5. (a) graphical representation of hRO differentiation and "early" dosing with RA receptor inhibitor AGN193019 (10µM) and recombinant FGF8 (100ng/ml) between d42-50, followed by "mid" AGN193109 pulse at d70-80 and "late" pulse at d120-130; (b-d) Relative mRNA expression of (b) RPC cell marker *VSX2* showing upregulation at d40 of differentiation compared with d0, (c) photoreceptor precursor markers, *RECOVERIN*, *CRX* and *AIPL1* showing photoreceptor specification from d40 of differentiation with expression further increasing to d90 and (d) cone precursor markers *RXRγ*, *THRβ* and *ONECUT1* significantly upregulated by d40, with plateauing expression from d60-70, indicating specification of cone precursors by this timepoint; (e) immunostaining of VSX2+ retinal progenitor cells in the neuroblastic layer (NBL) from d40 of differentiation, along with retinal ganglion cell (RGC) marker RBPMS in the inner nuclear layer (INL) from d60, OTX2+ cone-biased progenitor cells showing increasing cell numbers from d50, and RECOVERIN+ photoreceptor precursors from d70 predominantly localizing to the outer layers of developing hROs, and ONECUT1+ horizontal cell-biased retinal progenitors towards the inner layers of the organoid, alongside some OTX2+/RECOVERIN+ bipolar cells/mis-localized photoreceptor precursor cells; (f) Relative mRNA expression of mature cone markers *ARR3*, *OPSNSW*, *OPSNMW* and *OPSNLW* and (g) mature rod markers *RHODOPSIN*, *NRL* and photoreceptor marker *RECOVERIN* showing maturation of rods and cones by d150 of differentiation; (h) immunostaining of mature d150 retinal hROs stained for pan-photoreceptor marker RECOVERIN, with tight multilayered NRL+ rod nuclei in the outer nuclear layer (ONL), RHODOPSIN labelling the outer segments of rod photoreceptor cells and ARR3 labelling the cell bodies of a single apical layer of cone photoreceptor cells showing the inner segments, with a small number of S-OPSIN+ short wavelength cones, and M/L-OPSIN+ medium/long wavelength cones, with some RECOVERIN+ photoreceptors displaced to the INL. All qRT-PCR data shown as Log_2 fold change of CT values relative to d0 hESCs (n=3-5 pooled hROs per sample, N=3). Significance values determined by one-way

repeated measures ANOVA followed by Dunnett's test against control group, adjusting for multiple comparisons: *p value < 0.05, **p value < 0.01, ****p value < 0.0001, data are represented as mean ±SD. PSC – Pluripotent stem cell; E8 – Essential 8 media; E6 – Essential 6 media; PIM – Proneural induction media; RDM – Retinal differentiation media; ALT – Alternative RDM; RA – Retinoic Acid; AGN – AGN193109; NBL – Neuroblastic Layer; INL – Inner Nuclear Layer; ONL – Outer Nuclear Layer; IS – Inner segments; OS – Outer segments. Staining data representative of n=3 hROs from N=2 batches of differentiation, counterstained with DAPI (greyscale). Scale bars - 100μm.

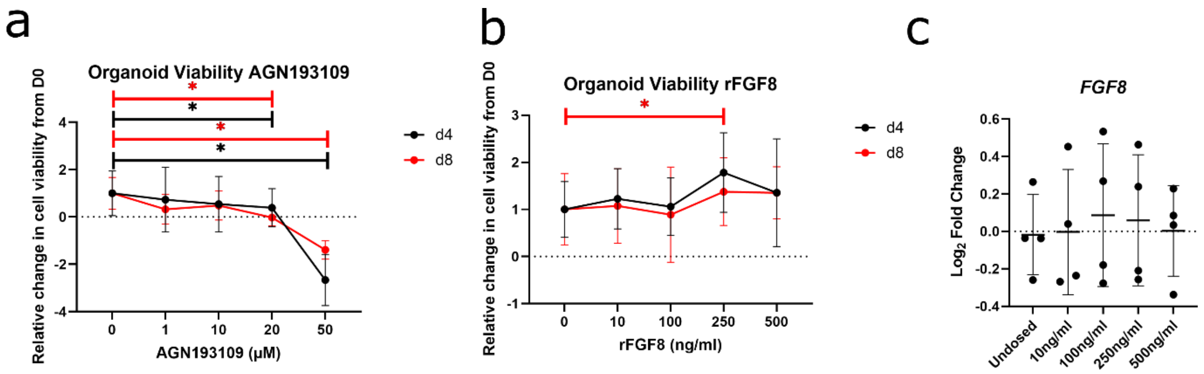

Fig.S4. Dose optimization of AGN193109 and recombinant FGF8 for hROs based on alamarBlue cell viability assay. Fluorescence was measured at 590nm (RFU) and change in reduction at d4 and d8, compared with d0 of dosing for each hRO, then relative change of treated compared with DMSO treated was calculated and plotted for (a) AGN193109 and (b) recombinant FGF8 (rFGF8) (n=5 from N=1 batch). (c) Dosing with rFGF8between d40-d50 lead to no significant change in *FGF8*, which is known to regulate its own expression Data shown as Log_2 fold change of CT values relative to PBS treated controls (5 pooled hROs per sample, N=4). Error bars - standard deviation. Significance values determined by one-way repeated measures ANOVA followed by Dunnett's test against control group, adjusting for multiple comparisons. **p* value<0.05.

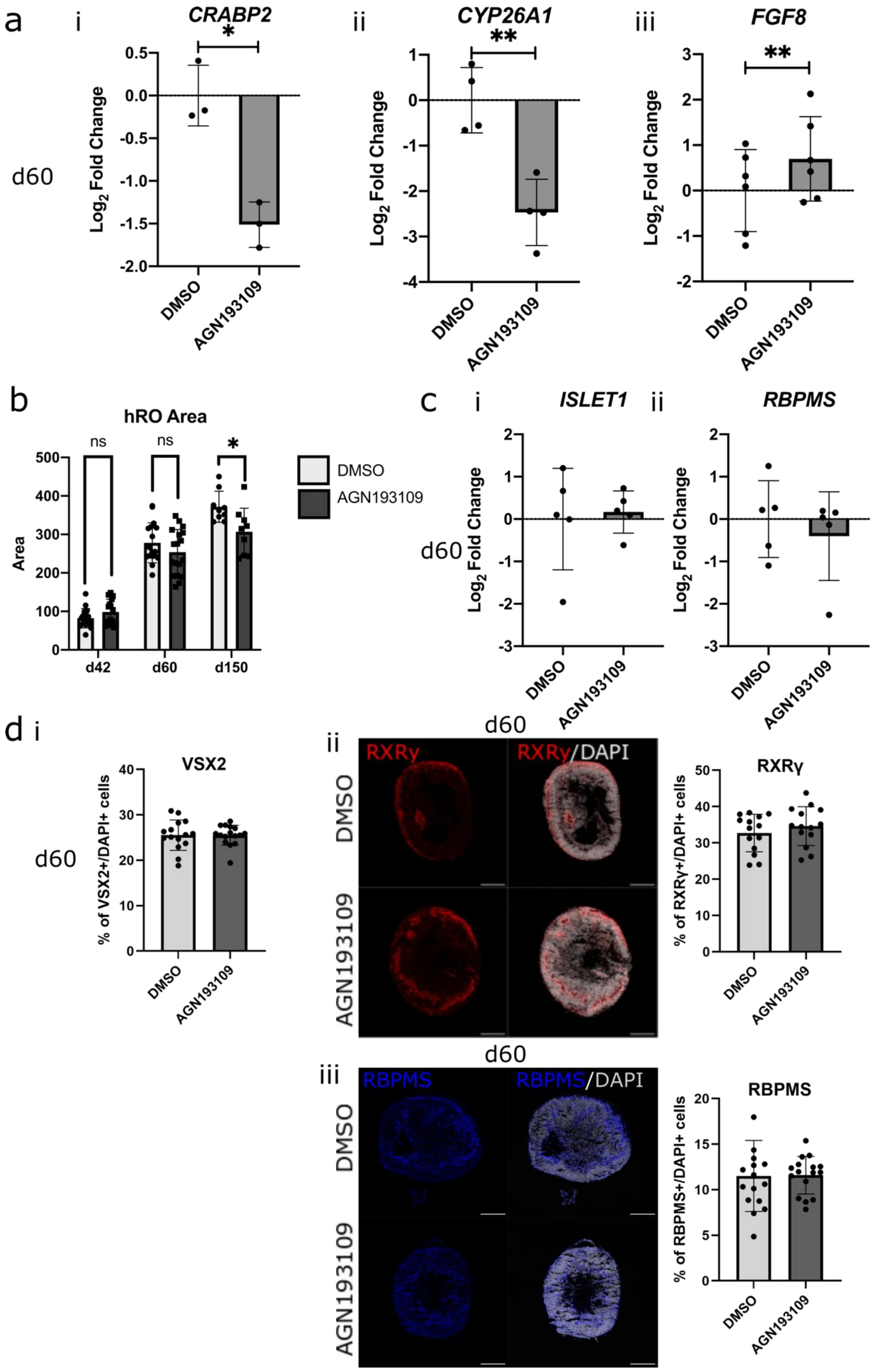
a
i
CRABP2
ii
CYP26A1
iii
FGF8
d60
Log2 Fold Change
DMSO
AGN193109
b
hRO Area
ns
Area
d42
d60
d150
c
ISLET1
RBPMS
d i
VSX2
% of VSX2+/DAPI+ cells
d60
RXRγ
RXRγ/DAPI
% of RXRγ+/DAPI+ cells
RBPMS/DAPI
% of RBPMS+/DAPI+ cells

83

Fig.S5. Dosing early hROs with RA receptor inhibitor AGN193109 does not alter retinal ganglion cell (RGC) marker or cone precursor marker expression at d60, related to Fig.6. (a-b) Dosing with RA receptor inhibitor AGN193109 between d42-d50 leads to (a) (i) a significant reduction in *CRABP2*, a proxy for circulating RA levels, ($t(2)=5.77$, $p=0.03$), (ii) a significant reduction in *CYP26A1,* indicating reduced cytoplasmic RA being catabolized ($t(3)=7.91$, $p=0.004$), and (iii) a significant increase in *FGF8*, a downstream effector of RA inhibition ($t(5)=5.51$, $p=0.003$); (b) Measurements of hRO area show no significant difference in size of treated/control samples at d42 (prior to dosing) (W=117, n=17, $p=0.24$) or d60 (10 days after dosing) (t(33)=1.28, p=0.21), although a significant difference in hRO growth was detected at d60 (Fig.6a), and a significant difference in hRO area was seen at d150 (t(16)=2.66, p=0.017). (c) No significant change in RGC marker *RBPMS* ($t(4)=1.02$, $p=0.37$) or RGC/bipolar/amacrine cell marker *ISLET1* (W=9, n=5, $p=0.31$). All data shown as Log_2 fold change of CT values relative to the mean of DMSO treated controls (n=5 pooled hROs per sample, N≥3, lines indicate paired samples of DMSO/AGN-dosed samples from the same batch of differentiation to control for batch-batch variation). Significance values determined by paired t-tests. (c) Immunostaining of AGN193109 dosed and DMSO dosed control hROs at d60 showing no significant change in the proportion of (i) RGC marker RBPMS (blue) cells ($t(30)=0.08$, $p=0.94$) (ii) cone precursor marker RXRγ (red) ($t(26)=0.96$, $p=0.35$) or (iii) RPC marker VSX2 (staining in Fig.6) ($t(29)=0.01$, $p=0.99$). Significance values determined by unpaired t-tests: *p value<0.05, n>13, data are represented as mean ±SD. Staining data representative of n>13 hROs from N=2 batches of differentiation, counterstained with DAPI (greyscale). Scale bars - 100μm.

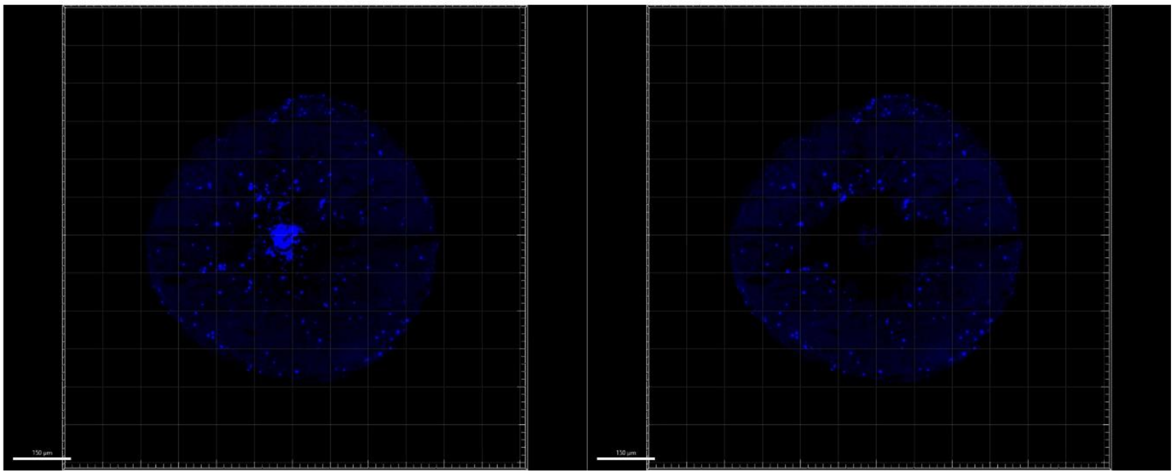

Fig.S6. Masking of necrotic core for CASPASE-3 staining quantification in IMARIS 10.0, related to Fig.6.

Supplementary Tables

Table S1 – Primary antibodies for IHC, relating to Fig.1-7, S1-3 and S5-6.

Target	Species	Manufacturer	Product code	RRID	Dilution
ALDH1A1	Rabbit	Abcam	ab23375	AB_2224009	1:200
ALDH1A3	Rabbit	Abcam	ab129815	AB_2937054	1:200
ARRESTIN-3	Goat	Novus	NBP1-37003	AB_2060085	1:100
CASPASE-3	Rabbit	Abcam	ab2302	AB_302962	1:100
CRX	Mouse	Abnova	H00001406-M02	AB_606098	1:800
KI67	Rabbit	Abcam	ab15580	AB_443209	1:100
M/L-OPSIN	Rabbit	Millipore	AB5405	AB_177456	1:100
NRL	Goat	R+D systems	AF2945	AB_2155098	1:100
ONECUT1	Mouse	Santa cruz	sc-13050	AB_2251852	1:100
OTX2	Goat	R&D Systems	AF1979	AB_2157172	1:50
RBPMS	Rabbit	PhosphoSolutions	1830	AB_2492225	1:150
RECOVERN	Rabbit	Chemicon	AB5585	AB_2253622	1:1000
RHODOPSIN	Mouse	Sigma	O4886	AB_260838	1:1000
RXRγ	Mouse	Santa Cruz	sc-365252	AB_10850062	1:200
S-OPSIN	Rabbit	Millipore	AB5407	AB_177457	1:200
SOX9	Rabbit	Sigma	AB5535	AB_2239761	1:200
VIM	Chicken	Novus	NB300	AB_922758	1:600
VSX2	Mouse	Santa Cruz	sc-365519	AB_10842442	1:200

Table S2 – Secondary antibodies for IHC, relating to Fig.1-7, S1-3 and S5-6.

	Manufacturer	Product code	RRID	Dilution
488 Donkey anti-rabbit	ThermoFisher	A21206	AB_2535792	1:400
488 Donkey anti-goat	ThermoFisher	A11055	AB_2534102	1:400
488 Donkey anti-mouse	ThermoFisher	A21202	AB_141607	1:400
546 Donkey anti-rabbit	ThermoFisher	A10040	AB_2534016	1:400
546 Donkey anti-goat	ThermoFisher	A11056	AB_2534103	1:400
647 Donkey anti-rabbit	ThermoFisher	A31573	AB_2536183	1:400
647 Donkey anti-goat	ThermoFisher	A21447	AB_2535864	1:400
647 Donkey anti-mouse	ThermoFisher	A31571	AB_162542	1:400
647 Donkey anti-chicken	ThermoFisher	A78952	AB_2921074	1:400

Table S3 – Primers used in this study for qRT-PCR analysis, related to Figs.5-7, S3 and S5.

Gene	Forward	Reverse	Universal Probe
AIPL1	GGAGAGGGAAATCGGCTCTT	TTCTCCTTGGTCTGCAGGTT	79
ALDH1A1	GTTAGCTGATGCCGACTTGG	CGCTCAACACTCCTTCGAAC	81
ALDH1A3	AAGAGGAGATTTTCGGGCCA	ACTTCAGGGCTTTGTCGAGA	66
ARR3	CACGGAGACTGTAGCTGCTA	GCTAGAGGCCAGGTTGGTAT	14
β-ACTIN	GCACCCAGCACAATGAAGAT	TCGTCATACTCCTGCTTGCT	63
CRX	GAGGCTGTGTCTTGTGCAAA	TCGAAGTCTTACCCCATCGG	12
CRABP2	CCCTGTAAGAGCCTGGTGAA	CCGTCATGGTCAGGATCAGT	93
CYP26A1	GACATGCAGGCACTAAAGCA	GGTAGAGCCCCAGGTAAGTG	5
FGF8	GAGACGGGCCTCTACATCTG	TGTACCAGCCCTCGTACTTG	67
FGFR1	CGGGACAGACTGGTCTTAGG	GGTTGGGTTTGTCCTTGTCC	53
FGFR2	CAGTCATCCTGTGCCGAATG	AACTGTTACCTGTCTCCGCA	71
FGFR3	TACTCCTTCGACACCTGCAA	ACTTCTGGGAGGCCAAGTAC	68
FGFR4	GGAGTCCCGGAAGTGTATCC	GTAGTCAATGTGGTGGACGC	71
GAPDH	TTGCCCTCAACGACCACTTT	TGGTCCAGGGGTCTTACTCC	77
ISLET1	TCTCCGGATTTGGAATGGCA	CATTTGATCCCGTACAACCTGAT	-
L-OPSIN	TGCATCATCCCACTCGCTAT	TTCCTTCTCTGCCTTCTGGG	5
M-OPSIN	CTACCTCCAAGTGTGGCTGG	GAATGCCAGGACCATCACCA	13
M/L-OPSIN	CTTCCACCCTTTGATGGCTG	AGTTTCGAAACTGCCGGTTC	-
NRL	AGAGCTGTGTGCAGAAGTCT	GCAGAAGTCGTCCAATCCAC	95
ONECUT1	CAGCGTCGAACTCTACATGC	ACTCCTCCTTCTTGCGTTCA	3
OTX2	ATTGCTAGAGCAGCCCTCAC	GGAGCAGTGGAACTTACAGC	-
RBPMS	CACTGCATGCCCAGATGC	CCCACAAGACAGATTGCAGC	-
RECOVERIN	GCTTCGATTCCAACCTCGAC	TTACCGTCCACGTCGTAGAG	78
RHODOPSIN	TGGGCCATTAAAAGCTCAGC	GTGGAAGCTGGCAGTTTCAA	77
RXRγ	CATGAAGAGGGAAGCTGTGC	TTCTGCCTCACTCTCAGCTC	82
SFRP1	GCCCGAGATGCTTAAGTGTG	GACACACCGTTGTGCCTTG	-
S-OPSIN	GCACTGTAGCAGGTCTGGTT	CAATGGTCCAGGTAGCCAGG	72
THRβ	ATTTCGTTCTCCGGTCCTGT	TCAAGTCTTGGACCAGGGAC	9
VSX2	TGGGGATGCACAAAAAGTCG	GCTCCATCTTGTCGAGCTTG	69

Supplementary Experimental Procedures

Human PSC culture and retinal differentiation

Established hESC line H9 (Wicell: WAe009-A, lot RB66492, passage (P)30) cells were maintained on Geltrex-coated (Gibco: A1413302) 6-well plates and fed daily with E8 media (Gibco, A1517001). Cells were passaged when 80% confluent in clumps using Versene (Gibco, 15040066) and plated 200,000 cells per well with 1:1000 Rock Inhibitor Y-27632 dihydrochloride (Tocris: 1254). Cells were cryopreserved with Knockout Serum Replacement (Thermo: 10828010) and 10% DMSO (Generon, DMSO-10) in liquid nitrogen.

Authentication of the Master Cell Bank by STR (Short Tandem Repeat) analysis was performed by WiCell, and confirmed exact match of the STR profile. Karyometrix testing was conducted on the working cell bank (P33), and the last culture passage (P64), with no major chromosomal changes detected at either stage. Pluripotency validation was performed for the working cell bank via flow cytometry of intracellular (Nanog, >70%; Oct3/4, Sox2; >80%) and extracellular markers (SSEA-1, <10%; SSEA-3, TRA-1-81, >80%) alongside positive immunocytochemistry staining for Nanog, Oct3/4 and Sox2. All experiments were performed on PSCs <15 passages after thawing from the working cell bank. During culture of both PSCs and hROs, regular mycoplasma and sterility testing was performed monthly.

For differentiation, human PSCs were maintained until 90-95% confluent, then on day 1 (d1) cultured in E6 media (Gibco, A1516401) for 2 days. Proneural induction media (PIM) was added for 40 days, changed every 2-3 days, consisting of Advanced DMEM/F12 (Gibco: 12634-010), N2 (Gibco 17502-048), Glutamine (Thermo Scientific:25030-024), Antibiotic-Antimycotic (Gibco: 15240-062), Non-essential amino acids (Gibco: 11140-035). Optic vesicles were manually excised from day 21 with 21G

needles and cultured in low-binding 96-well plates in retinal differentiation media (RDM), consisting of: DMEM (Gibco: 41965-039), F12 (Gibco: 31765-027), B27-vit A (Gibco: 12587-010), Antibiotic-Antimycotic (Gibco: 15240-062). At day 42, additional factors were added to RDM: 10% FBS (Gibco: 10500-064), 2mM Glutamax (Gibco: 35050-038), 100uM Taurine (Sigma: T4571). From day 70, media was changed to Alt70, consisting of: Advanced DMEM/F12 (Gibco: 12634-010), B27-vit A (Gibco: 12587-010), Antibiotic-Antimycotic (Gibco: 15240-062), 10% FBS (Gibco: 10500-064), 2mM Glutamax (Gibco: 35050-038), 100uM Taurine (Sigma: T4571) with 1uM retinoic acid added fresh. From day 90 media was changed to Alt90, consisting of: Advanced DMEM/F12 (Gibco: 12634-010), B27-vit A (Gibco: 12587-010), N2 (Gibco 17502-048), Antibiotic-Antimycotic (Gibco: 15240-062), 2mM Glutamax (Gibco: 35050-038), 100uM Taurine (Sigma: T4571), with 0.5uM retinoic acid added fresh (Biotechne: 0695/50).

Fetal retina/hRO sample preparation for Immunohistochemistry/ RNAscope™

For immunohistochemistry (IHC)/RNAscope™, human fetal samples were fixed for 1 day in 10% formalin before transferring to PBS. Samples were incubated overnight in 20% (w/v) sucrose (Merck: 84100), prior to cryo-embedding in OCT matrix (CellPath: 15212776). Tissue was cut at 10mM thickness, and mounted on Superfrost glass slides (Epredia: 10149870). hROs were washed with PBS and fixed for 1 hour in 4% PFA (Merck: P6148), followed by PBS wash and incubated overnight in 20% (w/v) sucrose, prior to cryo-embedding in OCT matrix. Tissue was cut at 10mM thickness, and mounted on Superfrost glass slides, air-dried overnight and frozen at -20°C.

RNAscope™ *in situ* hybridization

Samples were baked at 60°C for 30 minutes, postfixed with 4% PFA for 15 minutes at 4°C then dehydrated using an ethanol gradient, before 10-minute treatment with hydrogen peroxidase. Manual antigen retrieval was performed at 80°C for 5 minutes, then sections were pretreated with protease III for 30 minutes at 40°C. Sections were incubated with probes from the RNAscope™ probe catalogue Hs-CYP26A1-C1 (ACD Bio: 487741) and Hs-FGF8-C2 (ACD Bio: 415791-C2), or Human 3-plex positive (ACD Bio: 320861 – *POLR2a*/*PPIB*/*UBC*)/negative controls (ACD Bio: 320871 – DapB) for 3 hours at 40°C, before storing in 5xSSC (NaCl: Merck: S9625; Sodium Citrate: Thermo: 045556.30) overnight. Probes went through series of amplification steps, according to the manufacturer's protocol, before developing the signal and assigning a fluorochrome to each probe by incubating for 30 minutes with PerkinElmer cyanine 3/5 fluorophores, respectively. Following HRP blocking, slides were counterstained with VSX2 by incubating with primary antibody overnight, and secondary antibody Alexa-fluor 488 (**Table S2**)/DAPI (Invitrogen: D1306) for 2 hours, according to the IHC protocol and mounted in DAKO mounting medium (Agilent: S302380-2). Confocal images were taken using a Leica DM5500Q and processed in ImageJ. All image adjustments, including brightness, were applied uniformly to both experimental and control images.

Mean fluorescence intensity of *CYP26A2*/*FGF8* in each sample image (PM/ONH) as well as positive and negative controls for the corresponding channels was calculated using ImageJ (**Fig.S1b**). Fluorescence intensity of negative control was subtracted, to control for background noise, then intensity was normalized to positive control, to account for technical variation between samples. One-way ANOVA and Tukey's multiple comparison tests were performed in GraphPad Prism (v10.1.2).

Immunohistochemistry (IHC)

Cryosections were permeabilized for 1 hour at room temperature with PBS/0.1% Tween (Merck: P9416)/0.5% Triton X (Merck: T8787), then blocked for 2 hours at room temperature with PBS/0.1%

Tween/5% donkey serum/1% BSA (Merck: A9418). Primary antibodies with incubated overnight at 4°C (**Table S1**). Alexa Fluor® secondary antibodies (**Table S2**) were incubated for 1 hour at room temperature at 1:400 dilution, along with DAPI counterstain. Confocal images were taken using a Leica DM5500Q and processed in ImageJ. All image adjustments, including brightness, were applied uniformly to both experimental and control images. Analysis was performed using unpaired t-tests. Spot count analysis was performed using Imaris 10.0. For analysis of KI67 in human fetal tissue, the same length of region was selected at different regions of the same retina: the PM (identified by *CYP26A1*+ staining); the central region between the PM and nerve; the region the same distance as the PM from the periphery on the nasal side of the retina; and the periphery (**Fig.S2a**). For analysis of CAPSASE staining in hROs, necrotic core was masked to ensure only cell death in the neural retina cells was included (**Fig.S6**).

qRT-PCR gene expression analysis

For all qPCR assays, 3-5 hROs were pooled for each sample depending on collection timepoint (5 for d40-d90, 3 for d120-150). Samples were taken from independent experiments and a minimum of n=3 independent samples were used for analysis. RNA was extracted from hRO pellets using RNeasy Micro kit (Qiagen: 74004), according to manufacturer's instructions. Samples were eluted in 30ml and stored at -80°C. Concentration and RNA quality analyzed using a BioDrop. cDNA was synthesized using QuantiTect Reverse Transcription Kit (Qiagen: 205311), according to manufacturer's instructions. Quantitative RT-PCR was performed using Perfecta Low-ROX MasterMix (Quantabio: 95120-012) with custom primers listed in **Table S3** and probes from the Merck Universal Probe library or PowerTrack SYBR MasterMix (Applied biosystems: A46111) with ROX as passive reference. All samples were run in triplicate alongside 2 endogenous reference genes (*GAPDH* and *b-actin*), along with water and no RT negative controls. Comparative CT was calculated to determine relative gene expression as fold change

from either d0 PSCs or untreated controls. Treated/untreated samples were from the same batch of differentiation to control for batch-batch variation, and paired T-tests/one-way repeated measures ANOVA followed by Dunnett's test against control group was performed (adjusting for multiple comparisons).

hRO growth analysis

Growth of hROs was analyzed by measuring organoid area with ImageJ from brightfield images before and after dosing, from which % increase in area size calculated for each hRO.

Alamar Blue Viability assay

AlamarBlue utilizes resazurin which is reduced in presence of living cells and is non-toxic/cell permeable. Organoids were kept in separate wells of a 96-well plate and incubated for 4 hours with 10% AlamarBlue (Invitrogen: DAL1025) diluted in media, after which AlamarBlue was transferred to a new 96 well plate, organoids were washed 3 times with PBS and returned to media. Fluorescence of AlamarBlue at 590nm (RFU) was then measured using a plate scanner and % reduction calculated using:

$$Reduction_{AB} = \frac{RFU^{Exp} - RFU^{Neg}}{RFU^{100\%} - RFU^{Neg}} \times 100$$

In which $Reduction_{AB}$ is the percentage reduction of AlamarBlue, RFU^{Exp} is the Relative Fluorescence Units (RFU) for the experimental sample, RFU^{Neg} is the RFU for the Negative control, and $RFU^{100\%}$ is the RFU for the 100% reduced positive control.

To control for differences in hRO size, change in reduction from day 0 was calculated for each hRO:

$$\Delta Reduction = Reduction_{AB}^{Day\ X} - Reduction_{AB}^{Day\ 0}$$

Relative change in viability of treated organoids was normalized to DMSO treated controls:

$$\Delta Viability = \frac{\Delta Reduction^{Exp}}{\Delta Reduction^{Control}} \times 100$$

In which *ΔViability* is the change in viability from Day 0 in the experimental sample relative to the DMSO-dosed control sample

As hRO viability was significantly reduced upon dosing with 20µM AGN19309 but not 10µM, this concentration was used for all following experiments (**S4a Fig**). No reduction in viability was observed at any concentration of rFGF8 (**S4b Fig**). FGF8 can self-regulate its mRNA expression[56]. Consequently, expression of *FGF8* was assessed following dosing with rFGF8. No significant difference in *FGF8* expression was observed at any concentration, however, the largest mean difference was found at 100ng/µl (**S4b Fig**). Consequently, this concentration was used for all following experiments.
